# Supplementary material for: Worry perception and its association with work conditions among healthcare workers during the first wave of the COVID-19 pandemic: a web-based multimethod survey at a university hospital in Sweden
Source: BMJ Open. 2024 Feb 20;14(2):e080248. doi: 10.1136/bmjopen-2023-080248 (PMC10882285; doi:10.1136/bmjopen-2023-080248)
Supplement: Author's manuscript [file bmjopen-2023-080248.draft_revisions.pdf]

BMJ Open is committed to open peer review. As part of this commitment we make the peer review history of every article we publish publicly available.

When an article is published we post the peer reviewers' comments and the authors' responses online. We also post the versions of the paper that were used during peer review. These are the versions that the peer review comments apply to.

The versions of the paper that follow are the versions that were submitted during the peer review process. They are not the versions of record or the final published versions. They should not be cited or distributed as the published version of this manuscript.

BMJ Open is an open access journal and the full, final, typeset and author-corrected version of record of the manuscript is available on our site with no access controls, subscription charges or pay-per-view fees (http://bmjopen.bmj.com).

If you have any questions on BMJ Open's open peer review process please email info.bmjopen@bmj.com

BMJ Open

An online survey investigating the worry perceptions and the association with work conditions among healthcare workers during the first wave of the COVID-19 pandemic

Journal:	*BMJ Open*
Manuscript ID	bmjopen-2023-080248
Article Type:	Original research
Date Submitted by the Author:	25-Sep-2023
Complete List of Authors:	Alexiou, Eirini; Sahlgrenska University Hospital, Department of Forensic Psychiatry; University of Gothenburg<br>Wijk, Helle; University of Gothenburg; Sahlgrenska University Hospital, Department of Quality Strategies<br>Ahlstrom, Linda; University of Gothenberg<br>Degl' Innocenti, Alessio; Region Västra Götaland, Sahlgrenska University Hospital<br>Jonsdottir, Ingibjörg; Institute of Stress Medicine<br>Åkerström, Magnus; Institute of Stress Medicine
Keywords:	COVID-19, Health Workforce, OCCUPATIONAL & INDUSTRIAL MEDICINE

SCHOLARONE™
Manuscripts

I, the Submitting Author has the right to grant and does grant on behalf of all authors of the Work (as defined in the below author licence), an exclusive licence and/or a non-exclusive licence for contributions from authors who are: i) UK Crown employees; ii) where BMJ has agreed a CC-BY licence shall apply, and/or iii) in accordance with the terms applicable for US Federal Government officers or employees acting as part of their official duties; on a worldwide, perpetual, irrevocable, royalty-free basis to BMJ Publishing Group Ltd ("BMJ") its licensees and where the relevant Journal is co-owned by BMJ to the co-owners of the Journal, to publish the Work in this journal and any other BMJ products and to exploit all rights, as set out in our licence.

The Submitting Author accepts and understands that any supply made under these terms is made by BMJ to the Submitting Author unless you are acting as an employee on behalf of your employer or a postgraduate student of an affiliated institution which is paying any applicable article publishing charge ("APC") for Open Access articles. Where the Submitting Author wishes to make the Work available on an Open Access basis (and intends to pay the relevant APC), the terms of reuse of such Open Access shall be governed by a Creative Commons licence – details of these licences and which Creative Commons licence will apply to this Work are set out in our licence referred to above.

Other than as permitted in any relevant BMJ Author's Self Archiving Policies, I confirm this Work has not been accepted for publication elsewhere, is not being considered for publication elsewhere and does not duplicate material already published. I confirm all authors consent to publication of this Work and authorise the granting of this licence.

For peer review only

An online survey investigating the worry perceptions and the association with work conditions among healthcare workers during the first wave of the COVID-19 pandemic

Eirini Alexiou[1,2*], Helle Wijk[3,4,5], Magnus Akerstrom[6,7], Ingibjörg H. Jonsdottir[6,7], Alessio Degl' Innocenti[2,8], Linda Ahlstrom[3,9]

[1]Department of Forensic Psychiatry, Sahlgrenska University Hospital, Region Västra Götaland, Gothenburg, Sweden

[2]Center for Ethics, Law, and Mental Health (CELAM), Sahlgrenska Academy, University of Gothenburg, Gothenburg, Sweden

[3]Institute of Health and Care Sciences, Sahlgrenska Academy, University of Gothenburg, Gothenburg, Sweden

[4]Department of Quality Strategies, Sahlgrenska University Hospital, Region Västra Götaland, Gothenburg, Sweden

[5]Department of Architecture and Civil Engineering, Chalmers University of Technology, Gothenburg, Sweden

[6]Institute of Stress Medicine, Region Västra Götaland, Gothenburg, Sweden

[7]School of Public Health and Community Medicine, Institute of Medicine, Sahlgrenska Academy, University of Gothenburg, Gothenburg, Sweden

[8]Regionhälsan, Region Västra Götaland, Gothenburg, Sweden

[9]Department of Orthopaedics, Sahlgrenska University Hospital, Region Västra Götaland, Gothenburg, Sweden

*** Correspondence:**
Corresponding Author
eirini.alexiou@vgregion.se

Keywords: COVID-19; worry perceptions; pandemics; content analysis; healthcare workers (HCWs)

Abstract

Objective. In this study, we explored which factors influenced healthcare workers' (HCWs') worry perception and its association with their work situation during the first wave of the COVID-19 pandemic.

Methods. A web-based survey including multiple choice and open-ended questions was administered to all HCWs at a university hospital in Sweden who were working during the first wave of the COVID-19 pandemic in March-June 2020. HCWs (n=6484) from 69 departments fulfilled the study inclusion criteria and responded to the survey. Of them, 3532 replied to the open-ended questions.

Results. Four out of five HCWs reported high levels of worry of being infected or infecting others with the severe acute respiratory syndrome-related coronavirus (SARS-CoV-2), half of them daily. This finding could be further confirmed and explored with themes from the qualitative results: "Ambiguity of feeling safe and secure", "Being obliged to adapt to a new reality", and "Into the unknown". The themes consisted of six main categories and 15 sub-categories. The findings revealed that the two main drivers of worry perceived by HCWs were lack of personal protective equipment and fear of bringing the virus home to families and friends.

For peer review only

Conclusions.Worries of getting infected is common among HCWs during a crisis such as the COVID-19 pandemic. Several factors are raised that plausibly could minimize the negative effects of worry among the HCWs. Thus, effective preventive work plans should be created,promoted and communicated in order to miminzie the effects of such crises and to support HCWs. By focusing on effective communication, preparedness, including access to relevant protective equipment, and general support to the HCWs, the work environment and patient care could be sustained during a crisis such as the COVID-19 pandemic.'

Strengths and limitations of this study

1. The key strength of this study is transdisciplinary research cooperation, as several multi-disciplinary researchers participated in the implementation of the study and the analysis and interpretation of the data and findings.
2. For us working as a team when analyzing the data, the NVivo software program enhanced the quality of the analysis while data were more manageable and ordered and it was easy to compare and discuss the categories (Gerbic & Staecy 2005) and come to a consensus.
3. Additionally, the pandemic data collection was conducted during a relatively calm period between the first and second waves, which started around November 2020 in Sweden.
4. One study limitation is that the quantitative data was cross-sectional, where written answers were self-reported.
5. Another limitation is that the researchers could not ask follow-up questions to further develop and explore the answer.

1. Introduction

Worry refers to the thoughts, images, emotions, and actions of a negative nature in a repetitive, uncontrollable manner that result from a proactive cognitive risk analysis made to avoid or solve anticipated potential threats and their potential consequences (Borkovec et al., 2002). In other words, worry is a natural response to anticipated future problems (Bredemeier et al., 2008). When the threat is uncertain and continuous or when worry is not well calibrated to the actual threat, as during the coronavirus disease (COVID-19) pandemic, worry can become excessive and burdensome (Mertens et al., 2020).

The COVID-19 outbreak in China in December 2019 and Europe in February 2020 caused widespread worry, which when at healthy levels led to adaptive, protective behavioral changes. However, for some individuals, the pandemic outbreak led to excessive, maladaptive levels of worry. Experienced worries have been shown to be mainly related to perceived risks for loved ones, possible health problems related to COVID-19, and perceived social restrictions during the pandemic (Sobkow et al., 2020). High levels of worry during the COVID-19 pandemic were indicated in several surveys (Asmundson & Taylor, 2020a; McCarthy, 2020). In a cross-sectional study conducted in Thailand, 100% of the included healthcare workers (HCWs) reported worry and fear of COVID-19 related to infection control practices (Apisarnthanarak, 2020). Another cross-sectional study found that HCWs reported high levels of worry about getting infected and developing COVID-19, which indicates the great extent to which HCWs were highly worried about this scenario (Puci et al., 2020).

Both frontline and non-frontline HCWs are at risk of severe acute respiratory syndrome-related coronavirus 2 (SARS-CoV-2) infection and may therefore percive significant worries. Early on during the pandemic, COVID-19-pandemic-associated mental health distress became a notable problem for frontline HCWs (Tan et al., 2020; Lai et al., 2020) and a great number of HCWs became infected (Zhang, 2020, WHO, 2020b). Frontline HCWs, who took care of patients with COVID-19 daily, were at higher risk of developing worries compared to HCWs who did not

For peer review only - http://bmjopen.bmj.com/site/about/guidelines.xhtml

participate in the treatment of patients (Maunder et al., 2006). A systematic review report that increased level of potential exposure during the COVID-19 pandemic, as well as in previous coronavirus outbreaks, were shown to cause long-term worries (Carmassi et al., 2020).

In a previous study, we investigated the effects of the COVID-19 pandemic on the working environment of HCWs compared to the pre-pandemic situation (Jonsdottir et al., 2021). Results showed that worries about getting infected was one of several factors that influenced how HCWs perceived their work situation. In this study, we extend this knowledge by exploring which factors influenced HCWs' worry perception and the association of worry with perceived working conditions, such as work load and emotional support, during the first wave of the COVID-19 pandemic in Sweden. Understanding factors related to HCWs' worries could be used to design preventive measures in similar future situations and thereby improve HCWs' working conditions and prevent worry.

2. Materials and methods

This study is part of a larger research project investigating how the work environment and health of HCWs were affected by the COVID-19 pandemic. The current study utilizes a multi-method design comprising both qualitative and quantitative data from a survey containing multiple choice and open-ended answers. The first part consisted of self-reported quantitative data regarding worry about being infected and infecting others. The second part included participants' perceptions, which were captured in open-ended answers and analyzed with inductive content analysis. The last part investigated factors affecting the reported level of worry.

2.1 Setting

The study was conducted at a large university hospital in northern Europe, situated in western Sweden. The hospital provides emergency and basic care for the more than one million inhabitants in the region and offers highly specialized care for the 1.7 million inhabitants of West Sweden. During the pandemic, the hospital provided intensive care for patients with symptomatic COVID-19 disease.

2.2 Population and procedure

A web-based COVID-19 survey was administered to all hospital HCWs (n=17,914) regardless of having contact with patients (COVID-19 or in general) or having non-clinical work tasks (Jonsdottir et al., 2021). After excluding employees who were absent from work during the study period (n=1,399), 16,515 were eligible for study participation (Figure 1).

During the first week of September 2020, an invitation to participate was sent by e-mail including a link to an anonymous survey. One reminder was provided during the last week of September 2020. The possibility to answer the survey was approximately 5 weeks. Study participants were asked to keep in mind how they perceived their working conditions during the intensive period of the pandemic in the spring of 2020 when answering the questionnaire.

2.3 Ethical considerations

The study was approved by the Swedish Ethical Review Authority (ref. 2020-04771) and participants provided informed consent. The study was conducted in compliance with the Helsinki Declaration and the General Data Protection Regulation (EU) 2016/679.

2.4 Survey and outcome measures

The survey was designed to be completed in 10-20 minutes. Demographic items included age, gender, organizational affiliation, professional role, specialist training, working hours (daytime, evening, night shifts, or varied hours), and eleven single-items regarding work conditions addressing work demands, support, recovery, and engagement were collected (Jonsdottir et al., 2021). Additional items about work placement during the pandemic, worries about getting infected, and access to personal protective equipment (PPE) were included. Participants were

For peer review only - http://bmjopen.bmj.com/site/about/guidelines.xhtml

additionally asked to reflect in an open-ended question about their positive and negative experiences of the pandemic during the first wave.

2.5 Qualitative analysis

To capture new insights and knowledge of the phenomenon of worry of HCWs during the first wave of COVID-19, we used an inductive content analysis of the response to the open-ended question, performed in three phases: 1) preparation phase, 2) organizing phase, and 3) reporting phase (Elo & Kyngäs, 2008; Elo et al., 2014). In the first phase, the first and last authors carefully read the text separately, to obtain their own comprehensive understanding. Thereafter, they each selected the unit of analysis and condensed the data. In the second phase, data were coded, grouped, and sub-categories, main-categories, and themes were created. The data analysis included an iterative process, with regular reconciliations and discussions, where data were compared for similarities, differences, and patterns, to enhance the confirmability of the findings. Working strategically prevents researcher bias and ensures data coding is externally heterogeneous and internally homogenous (Patton, 2014). Computer-assisted qualitative data analysis software, Nvivo 12 (QSR International), was used to aid in data organization. Trustworthiness was ensured by following the steps from the data collection to the reporting of the results as stated by Elo *et al.* (2014). Analysis also followed the Consolidated criteria for reporting qualitative research (COREQ), a 32-item checklist for interviews (Tong et al., 2007).

2.6 Quantitative analysis

Individuals without informed consent (n=83), missing data on all work environment items (n=21), or missing an organizational affiliation (n=211) were not included in the analysis. Excluded individuals were evenly distributed among departments and professional roles. One administrative department had limited respondents (n=7) and was excluded due to the risk of identification of individuals. The percentage of HCWs with a strong worry for being infected and for infecting others during the first wave of the COVID-19 pandemic was summarized descriptively.

To assess the impact of age, experiencing lack of PPE, being redeployed to another department, caring for patients with COVID-19, and perceived working conditions on HCWs worry of being infected or infecting others, mixed-effects models (Proc Mixed in SAS version 9.4; SAS Institute, USA) were used with these measures as fixed effects (nested within departments) and departments as a random effect. Hypothesis testing for fixed and random effects was performed using Wald tests and likelihood ratio tests, respectively. Statistical significance was set at $p<0.05$ and two-sided confidence intervals were used.

3. Results

There were 6,484 HCWs, from 69 departments at the hospital, who responded the survey and fulfilled the inclusion criteria for this study,. Of them, 3,532 (54%) replied to the open-ended questions, which generated about 216 pages of A4 text.

Most of the responders were women (83%) and 30% were employed as registered nurses, 21% as assistant nurses, 10% as physicians, 12% as administrative staff, and the remaining belonged to other occupations such as midwives, psychologists, managers, technicians, and physiotherapists. The age distribution was 12% of participants who were ≤ 29 years old, 22% aged 30-39 years, 23% aged 40-49 years, 27% aged 50-59 years, and 16% who were ≥ 60 years.

Percentage of HCWs with a strong worry about being infected and infecting others during the first wave of the COVID-19 pandemic

A total of 28% and 35% of the HCWs experienced a daily, or more than daily, strong worry about being infected or infecting others, respectively (Figure 2). There was a high correlation between expressing a strong worry about being infected and about infecting others ($r=0.67$, $p<0.001$).

For peer review only - http://bmjopen.bmj.com/site/about/guidelines.xhtml

HCWs worries during the first wave of the COVID-19 pandemic - qualtative analysis

The analysis of the qualitative data resulted in three themes: "Ambiguity of feeling safe and secure", "Being obliged to adapt to a new reality", and "Into the unknown". The themes consisted of a total of six main categories and 15 sub-categories (Table 1).

***Theme:* Ambiguity of feeling safe and secure**

This theme contains conceptions relating to the perceived concern of protection against the virus when exposed. This includes having or lacking access to PPE against COVID-19 such as face masks and/or respirators, face shields/goggles, and protective clothing, as well as conceptions about being able to use the available PPE, and being able to be tested for SARS-CoV-2 when experiencing symptoms. The theme consists of two main categories, "Accessibility" and "Communication", which are both related to reasons the HCWs perceived their ability to protect themselves and patients from encountering the virus as either good or poor.

The main category, "Accessibility", covers three subcategories: availability of PPE and testing for SARS-CoV-2 infection, priorities over testing and using PPE, and training in the proper use of PPE. HCWs described feeling forced to prioritize PPE use when the availability of PPE was limited. In many cases, HCWs lacked access to PPE when needed. Furthermore, they sometimes lacked the opportunity to train and become confident in the proper use of PPE, including when to use it. Results demonstrated that HCWs did not always lacked access to PPE and testing due to material shortage but also due to prioritization decisions and to different time schedules and priorities between departments. In general, HCWs experienced that those who were not working in the frontline, were not always allowed to use PPE or be tested for SARS-CoV-2 infection.

"The positive has been that there is protective equipment to use and that all patients are tested regularly." – P33, Assistant nurse, Geriatrics department

"The negative was that I experienced a few re-evaluations of the need for protective equipment when it started to run out. It was in the middle of that period when we had the most to do and no one knew how it would end. To then claim that COVID-19 was not contagious if you had a visor (which happened to be the protective equipment we had in stock and could be reused) felt disrespectful and completely illogical." – P66, Medical resident, Geriatrics department and Emergency department

The second main category, "Communication", contains conceptions about how clarity in information as well as constantly changing routines and decisions, impacted HCWs' feelings of being able to protect themselves and others from the virus.

"Very unclear sometimes regarding protective equipment, especially at the beginning of the pandemic when routines regarding protective equipment were changed very often, created an uncertainty." – P2, Registered nurse, Pediatrics department

"Lack of clarity in routines when it came to the use of protective equipment in different situations." – P42, Registered nurse, Radiology department

***Theme:* Being obliged to adapt to a new reality**

This second theme contains perceptions relating to feelings of inadequacy, despite doing one's best, and feelings of sadness when being incapable of providing sufficiently good care. These feelings also addressed handling the challenges in patient care that came with the COVID-19 pandemic, e.g., to have to prioritize daily patient care and make difficult decisions, while feeling scared and sad about being unable to deliver good quality care to patients.

This theme consists of two main categories, "Competence" and "Mission". The first main category, "Competence", covers two subcategories, "Learning" and "Workshifting". These subcategories are related to the feeling of having relevant clinical knowledge (or not) and the skills needed to perform new tasks at the new working place to which HCWs were assigned during the pandemic.

For peer review only - http://bmjopen.bmj.com/site/about/guidelines.xhtml

"Being forced to do a work for which you lack training and experience in a completely new workplace without receiving training and without a work description" – P85, Registered nurse, Intensive care unit

"Workshifting, which we fought for a long time to happen, now happened quickly and smoothly". – P25, Healthcare administrative secretary, medical secretary, Emergency department

The subcategory "Competence" was also related to the feeling of having had the opportunity to learn the skills needed to fight against the virus but also to adapt to the demands of digital transformation and to adopt new technologies by attending courses and other educational and training programs. HCWs report mixed perceptions of adapting to the new skills and tasks, leading in some cases to increased worries and concern.

"What amazing people who work in health care! To adapt quickly to new guidelines, new care, new workplace, colleagues, etc." – P146, Assistant nurse, Hand surgery department

The second main category, *"Mission"*, includes two subcategories, *"*Care priorities" and *"*Patient outcomes*"*. These were mostly related to the outcomes of one's own personal efforts and the feeling of being able to protect patients from getting infection and care for patients to save their lives, as well as making priorities and having enough time to spend with the patients in need.

"I felt inadequate towards the patients. I could not always provide as good care as desired due to high workload." – P24, Registered nurse, Infectious diseases department

"The most negative thing was all the patients who died without having someone with them. Neither relatives nor HCWs. It feels hard to have been a part of that. Overall, what was experienced as difficult is that the patients with COVID (or suspected COVID) got stuck, their care was not always good." – P31, Registered nurse, Infectious diseases department

***Theme:* Into the unknown**

The third theme contains conceptions relating to the worries about the COVID-19 pandemic and potential outcomes. It consists of two main categories, "Challenges" and "Social interaction". "Challenges" covers two subcategories, "Infection exposure" and "What tomorrow brings". These subcategories are related to worries about being exposed to the COVID-19, of oneself becoming infected, of transmitting the virus to someone else, or that patients infect each other. The second sub-category describes an emotional state caused by feared, unpredictable consequences on personal somatic health. No one knew who would be infected and the outcome of infection for any particular individual.

"I was surprised how scared I was, I'm not a scared person, but this really scared me, and it didn't feel very safe when some of my colleagues were infected at the departments where they worked. It felt good that the hospital locked its doors and that there were guards." – P21, Administrative assistant, Health and rehabilitation department

The second main category, "Social interaction", covers three subcategories: "Socialising with family and friends", "Interaction with colleagues", and "Patient interaction". Reported worries were related to the emotional state due to feared consequences regarding those with whom one was sociliazing or interacting, thereby affecting the health of loved ones, colleagues, and patients not yet infected.

"Negative: Strong concern that my relatives would get COVID-19." – P104, Assistant nurse, Intensive care unit

Factors affecting the level of worry among HCWs

The worry of being infected or infecting others decreased with age ($p=0.002$ and $p<0.001$, respectively) and increased when experiencing a lack of PPE ($p<0.001$, respectively), being redeployed to another department ($p=0.03$ and $p=0.05$, respectively) and when caring for patients with COVID-19 ($p<0.001$, respectively).

For peer review only - http://bmjopen.bmj.com/site/about/guidelines.xhtml

To further disentangle the effect of the factors identified in the qualitative analyses the associations between worry about being infected and caring for patients with COVID-19 in combination with access to PPE when needed and perceived working conditions (i.e., access to emotional support and a reasonable amount of work) were investigated. Results show that caring for patients with COVID-19, especially in combination with a perceived lack of PPE, was associated with a higher percentage of HCWs reporting a daily strong worry about being infected (Figures 3 and 4). Simultaneously, a lack of emotional support (Figure 3) and a reasonable amount of work (Figure 4) led to higher levels of strong worry.

4. Discussion

4.1 Main findings The quantitative results showed that four out of five HCWs reported high levels of worry, half of them daily, which could be further confirmed and explored with themes from the qualitative results.These worries decreased among participants who were supported by accessible PPE and clear communication about routines and how to protect oneself . Worries related to a lack of feeling safe, due to lack of PPE while working, is in line with results from previous studies (The Lancet, 2020). The rationale for being worried was related to the rapid spread of COVID-19, its strong contagion factor, and lethality in severe cases, without any specific medication for treatment (Koffman et al., 2020). Nevertheless, this is surprising considering the knowledge within the healthcare sector concerning strategies to educate and comfort HCWs in infection control. In the Swedish healthcare setting, there is a long tradition of a strong focus on adherence to hygiene routinesand knowledge regarding how the spread of infection prevention. (Megeus et al., 2015; Wikstrom et al., 2019).
However, there is evidence that repeated training on the proper use of PPE (Holthof et al., 2020), together with regular updates on COVID-19 pandemic (Hofmeyer et al., 2021), is successful in reducing worry among HCWs. So if the hospital would have offered specific and intense training in infection prevention, regular COVID-19 testing, and contact tracing to HCWs on an early stage, this might have decreased the HCWs' worries regarding unsafety and strengthened the impression of the management supporting and empowering employees by taking such actions. On the other hand, a survey from Thailand found that feelings of worry resulted in higher compliance with PPE and infection prevention practices (Apisarnthanarak, 2020), as well as patient safety risk behavior, thereby improving patient outcomes. Pointing at that managers should proactively seek information from recent evidence on the most effective ways to support and enhance HCWs' safety in order to alleviate and validate the concerns of HCWs,. So in sum, the results from our and earlier studies' indicate that the organization must be prepared and establish strategies and workplans for pandemics and other crises.
Another recommended strategy for managers, is to focus on early signs of individual HCW's worries and to offer support from psychological services (Puci et al., 2020) and to work closely with their employees, express empathy, gratitude, and prudence for HCWs' valuable efforts (Hofmeyer and Taylor, 2021), and confirm their worries when the situation is beyond one's control (Radbruch, 2020).
The increased burden on HCWs during crises needs to be adjusted by relocating internal and external flow capacity, and by training and strengthening the available workforce (Holthof et al., 2020). Which skills and competencies that new crises may demand differs and it is therefore complicated and even impossible to know what may be needed when going into the unknown. Nevertheless, there are ways for organizations to prepare. To adjust to a new reality there is a need to ensure adequate staffing levels with the right competence and clinical knowledge skills, and with appropriate workload to have the confidence to succeed with new tasks without compromising safety, quality of care, and patient outcomes, even though the individual employee might have to work at a different workplace or undergo workshifting.

For peer review only - http://bmjopen.bmj.com/site/about/guidelines.xhtml

Times consumed on different work tasks vary, and the schedule does not take into consideration extra time for donning PPE, extra rest days, maintaining education training, and training new colleagues. As reported here, HCWs adapted and learned new skills promptly, and were proud and impressed by that. For this to work, organizations need to be able to better provide both emergency and planned care during and after crises and balancing structures are needed that strengthen organizational resilience (Rangachari, 2020). Resilience at the individual level has limited impact unless the work to strengthen the work-place also infiltrates the team and organizational levels. Rangachari and Woods (2020) therefore highlight the importance of viewing organizational resilience as a system developed by managers to learn from the work of individual employees with a focus on proactivity, adaptation, and recovery.

The higher level of worry among HCWs motivated them to adjust to a new, unpredictable reality. One way to address this is through the implementation of effective risk communication programs to increase awareness of the risks associated with the pandemic and promote the adoption of appropriate preventive measures. Healthcare organizations and policymakers need to prioritize the implementation of effective risk communication strategies to promote safety among HCWs. However, there are ways to be prepared for crises in order to improve the work environment including increase safety, and to accept that it is not a failure to admit that everything is unknown, and that one feels very worried (Koffman et al., 2020). Developing strategies for managing worries can further support building resilience in the face of uncertainty and hinder the consequences of professional worries on work quality and reduce the negative impact on patient outcomes (Di Trani, 2021). In line with a multi-center study (Mertens, 2020), our study identified that the most frequently reported concern was for others to be infected by SARS-CoV-2 and worry about the health of loved ones. Notably, respondents in the multi-center study expressed various worries, including the impact of COVID-19 on the healthcare system, the economy, society, work loss, and changes in daily routines. Our study supports these findings and demonstrates that worries in all areas were referred to as factors that have an impact on the individual, organizational, and societal levels.

4.2 Methodological discussion

An essential step in the qualitative data analysis process involved categorizing the results. This process went beyond merely grouping data that appeared similar or connected. Rather, it involved a deliberate effort to gain a deeper understanding of the phenomena under investigation by classifying the data into meaningful categories (Elo & Kyngäs, 2005). In this study, the authors' pre-understandings of the studied phenomena differed; the first, second, and last authors are registered nurses, who participated in the first part of the analysis. The variability in experience can be explained by the professions' respective scopes of practice and competencies in relation to their worries.

Quality criteria and recommendations in the form of checklists have been synthesized by multiple publications for researchers. However, there have been raised concerns that checklists may suppress the diversity and multiplicity of practices within the qualitative paradigm, thus researchers must be aware and open-minded. As an alternative, throughout this study, best practice guidelines were applied to achieve and assess methodological rigor and research quality. Guidelines by Elo and Kyngäs (2005) and the COREQ checklist (Tong et al., 2007) were followed to uphold quality. We used data saturation to describe the achievement of a sufficient sample size, and ethical conduct was maintained throughout the research process. The methodology was acknowledged to be limited by researcher bias, which was minimized through predetermined plans. Multiple sources of data were used to optimize accuracy in data collection and analysis processes, resulting in credible and confirmable results (Johnson et al., 2020).

4.3 Conclusions

For peer review only

For peer review only - http://bmjopen.bmj.com/site/about/guidelines.xhtml

Overall, this study raises several factorsthat plausibly could help minimizing the negative impact of the COVID-19 pandemic on HCWs' worries. Developing strategies for managing worries during crises should be created and promoted in order to support and strengthen HCWs. By focusing on effective communication, preparedness, including access to relevant protective equipment, and general support to the HCWs, the work environment and patient care could be sustained during a crisis such as the COVID-19 pandemic.

5. List of abbreviations

HCWs Healthcare workers

SARS-CoV-2 Severe acute respiratory syndrome-related coronavirus

Covid-19 CoronaVirus Disease-2019

6. Declarations

Ethics approval and consent to participate

The studies involving human participants were reviewed and approved by the Swedish Ethical Review Authority (Ref. 2020-04771). The patients/participants provided their written informed consent to participate in this study.

7. Conflict of Interest

The authors declare that the research was conducted in the absence of any commercial or financial relationships that could be construed as a potential conflict of interest.
The authors declare that they have no competing interests.

8. Funding

This research received no specific grant from any funding agency in the public, commercial or not-for-profit sectors.

9. Authors' contributions

EA and LA designed the study, collected the data, analyzed the qualitative data, and drafted and revised the manuscript. HW designed the study, collected the data, and drafted and revised the manuscript. MA designed the study, collected the data, made the quantitative statistical analyses and revised the manuscript. IJ and AD'I designed the study, collected the data, and revised the manuscript. All authors contributed to the article and approved the submitted version.

10. Acknowledgements

We would like to provide our sincere thanks to the employees of the University Hospital for their time responding to the survey.We would also like to thank Flora Cassiano and Sandra Pettersson for their valuable help with the administration of the survey and with the data analysis. And lastly, we would also like to thank the Human Resources Department at the University Hospital, particularly Camilla Nilsson and Berit Roos Holmquist, for their collaboration and support in the development of the survey.

11. References

Tong A., Sainsbury P., Craig J. Consolidated criteria for reporting qualitative research (COREQ): a 32-item checklist for interviews and focus groups, International Journal for Quality in Healthcare. December 2007, Volume 19, Issue 6, Pages 349–357, https://doi.org/10.1093/intqhc/mzm042

Apisarnthanarak, A., Apisarnthanarak, P., Siripraparat, C., Saengaram, P., Leeprechanon, N., & Weber, D. J. Impact of anxiety and fear for COVID-19 toward infection control practices among Thai healthcare workers. Infection Control & Hospital Epidemiology. 2020;41(9), 1093-1094.

Borkovec T.D. Clinical Psychology: Science and Practice. 2002;9, 76–80.

Bredemeier K., Berenbaum H. "Intolerance of uncertainty and perceived threat". Behaviour Research and Therapy. 2008;46 (1): 28–38. doi:10.1016/j.brat.2007.09.006. PMID 17983612

For peer review only - http://bmjopen.bmj.com/site/about/guidelines.xhtml

Carmassi C., Rossi A., Pedrinelli V., Cremone I.M., Dell'Oste V., Stratta P., Bertelloni C.A., Dell'Osso L. PTSD in the aftermath of a natural disaster: what we learned from the Pisa-L'Aquila Collaboration Project. J. Psychopathol. 2020;26(1):99–106. doi: 10.36148/2284-0249-377

Carter N., Bryant-Lukosius D., DiCenso A., Blythe J., Neville A.J. Oncol Nurs Forum. 2014;41(5):545–47.

Elo, S., & Kyngäs, H. The qualitative content analysis process. Journal of Advanced Nursing. 2008; 62(1), 107-115. doi: 10.1111/j.1365-2648.2007.04569.x

Elo, S., Kääriäinen, M., Kanste, O., Pölkki, T., Utriainen, K., & Kyngäs, H. Qualitative content analysis: A focus on trustworthiness. SAGE open. 2014; 4(1), 2158244014522633.

Asmundson G.J.G., Taylor S. COVID-19phobia: Fear and the 2019-nCoV outbreak. Journal of Anxiety Disorders, 2020; 70, p. 102196, 10.1016/j.janxdis.2020.102196

Mertens G., Gerritsen L., Duijndam S., Salemink E., Engelhard I.M. Fear of the COVID-19 (COVID-19): Predictors in an online study conducted in March 2020, Journal of Anxiety Disorders. March 2020; Volume 74, 2020, 102258, ISSN 0887-6185, https://doi.org/10.1016/j.janxdis.2020.102258.

Gerbic P. & Stacey E. A purposive approach to content analysis: designing analytical frameworks. Internet and Higher Education. 2005; 8, 45–59.

Hofmeyer, A., & Taylor, R. Strategies and resources for nurse leaders to use to lead with empathy and prudence so they understand and address sources of anxiety among nurses practising in the era of COVID-19. Journal of clinical nursing. 2021; 30(1-2), 298-305.

Holthof, N., & Luedi, M. M. Considerations for acute care staffing during a pandemic. Best practice & research. Clinical anaesthesiology. 2021; 35(3), 389–404. https://doi.org/10.1016/j.bpa.2020.12.008

J. McCarthy. U.S. COVID-19 concerns surge, government trust slides (2020) https://news.gallup.com/poll/295505/COVID-19-worries-surge.aspx

Johnson, J. L., Adkins, D., & Chauvin, S. A Review of the Quality Indicators of Rigor in Qualitative Research. American journal of pharmaceutical education. 2020; 84(1), 7120. https://doi.org/10.5688/ajpe7120

Jonsdottir I.H, Degl'Innocenti A., Ahlstrom .L, Finizia C., Wijk H., Åkerström M. A pre/post analysis of the impact of the COVID-19 pandemic on the psychosocial work environment and recovery among healthcare workers in a large university hospital in Sweden. J Public Health Res. 2021 Jul 14;10(4):2329. doi: 10.4081/jphr.2021.2329. PMID: 34278769; PMCID: PMC8715269.

Koffman, J., Gross, J., Etkind, S. N., & Selman, L. Uncertainty and COVID-19: how are we to respond?. Journal of the Royal Society of Medicine. 2020; 113(6), 211-216.

Lai J., Ma S., Wang ., et al. Factors Associated With Mental Health Outcomes Among Healthcare Workers Exposed to COVID-19 Disease 2019. JAMA Netw Open. 2020;3(3):e203976. doi:10.1001/jamanetworkopen.2020.3

Maunder R.G., Lancee W.J., Balderson K.E., Bennett J.P., Borgundvaag B., Evans S., Fernandes C.M.B., Goldbloom D.S., Gupta M., Hunter J.J., McGillis Hall L., Nagle L.M., Pain C., Peczeniuk S.S., Raymond G., Read N., Rourke S.B., Steinberg R.J., Stewart T.E., VanDeVelde-Coke S., Veldhorst G.G., Wasylenki D.A. Long-term psychological and

For peer review only - http://bmjopen.bmj.com/site/about/guidelines.xhtml

occupational effects of providing hospital healthcare during SARS outbreak. Emerg. Infect. Dis. 2006;12(12):1924–1932. doi: 10.3201/eid1212.060584.

Megeus V., Nilsson K., Eriksson B., Karlsson J., Andersson Erichsen A. Hand hygiene and aseptic techniques during routine anesthetic care - observations in the operating room. Antimicrobial Resistance and Infection Control. 2015;4(5).

Megeus, V., Nilsson, K., Karlsson, J., Eriksson, B. I., & Andersson, A. E. Hand contamination, cross-transmission, and risk-associated behaviors: an observational study of team members in ORs. Aorn Journal. 2015; 102(6), 645-e1.

Puci, M. V., Nosari, G., Loi, F., Puci, G. V., Montomoli, C., & Ferraro, O. E. Risk Perception and Worries among Healthcare Workers in the COVID-19 Pandemic: Findings from an Italian Survey. Healthcare (Basel, Switzerland). 2020; 8(4), 535. https://doi.org/10.3390/healthcare8040535

Rangachari, P., & L. Woods, J. Preserving organizational resilience, patient safety, and staff retention during COVID-19 requires a holistic consideration of the psychological safety of healthcare workers. *International journal of environmental research and public health*. 2020; *17*(12), 4267. https://doi.org/10.3390/ijerph17124267

Shapiro, S. S., & Wilk, M. B. An Analysis of Variance Test for Normality (Complete Samples). Biometrika. 1965; 52(3/4), 591–611. https://doi.org/10.2307/2333709

Sobkow, A., Zaleskiewicz, T., Petrova, D., Garcia-Retamero, R., & Traczyk, J. Worry, Risk Perception, and Controllability Predict Intentions Toward COVID-19 Preventive Behaviors. Frontiers in psychology. 2020; 11, 582720. https://doi.org/10.3389/fpsyg.2020.582720

Tan, B., Chew, N., Lee, G., Jing, M., Goh, Y., Yeo, L., Zhang, K., Chin, H. K., Ahmad, A., Khan, F. A., Shanmugam, G. N., Chan, B., Sunny, S., Chandra, B., Ong, J., Paliwal, P. R., Wong, L., Sagayanathan, R., Chen, J. T., Ng, A., Sharma, V. K. Psychological Impact of the COVID-19 Pandemic on Healthcare Workers in Singapore. Annals of internal medicine. 2020; 173(4), 317–320. https://doi.org/10.7326/M20-1083

The Lancet. COVID-19: protecting health-care workers. Lancet. 2020 Mar 21;395(10228):922. doi: 10.1016/S0140-6736(20)30644-9. PMID: 32199474; PMCID: PMC7138074.

WHO (2020b). COVID-19 disease (COVID-19) pandemic [Online]. 2020; Available at: https://www.who.int/emergencies/diseases/novel-COVID-19 2019?gclid=EAIaIQobChMI7dXri7j36gIVWCUrCh2lLg2TEAAYASAAEgKwIvD_BwE (Accessed December 12, 2020).

Wikstrom E., Dellenborg L., Wallin L., Gillespie B.M., Erichsen Andersson A. The Safe Hands Study: Implementing aseptic techniques in the operating room: Facilitating mechanisms for contextual negotiation and collective action. American Journal of Infection Control. 2019;47(3):251-7.

Zhang Y., Ma Z.F. Impact of the COVID-19 Pandemic on Mental Health and Quality of Life among Local Residents in Liaoning Province, China: A Cross-Sectional Study. Int J Environ Res Public Health. 2020 Mar 31;17(7):2381. doi: 10.3390/ijerph17072381. PMID: 32244498; PMCID: PMC7177660.976

12. Data Sharing Statement

The raw data supporting the conclusions of this article will be made available by the authors, without undue reservation.

For peer review only

13. Tables and figures

Table 1. Subcategories, main categories, and themes concerning worry perceptions among healthcare workers during the first wave of the COVID-19 pandemic onset in spring 2020.

Subcategories	Main categories	Themes
Availability of personal protective equipment and testing for Covid-19	Accessibility	Ambiguity of feeling safe and secure
Priorities over testing and using protective equipment		
Training in proper use of personal protective equipment		
Information	Communication	
Routines		
Decisions		
Learning	Competence	Being obliged to adapt to a new reality
Workshifting		
Care priorities	Mission	
Patient outcomes		
Infection exposure	Challenges	Into the unknown
What tomorrow brings		
Socializing with family and friends	Social interaction	
Patient interaction		
Interaction with colleagues		

For peer review only

For peer review only - http://bmjopen.bmj.com/site/about/guidelines.xhtml

Figure 1. Flow chart showing the total number of hospital health care workers, the number who were eligible to participate, the response rate, and the final number of responses included in the analysis.

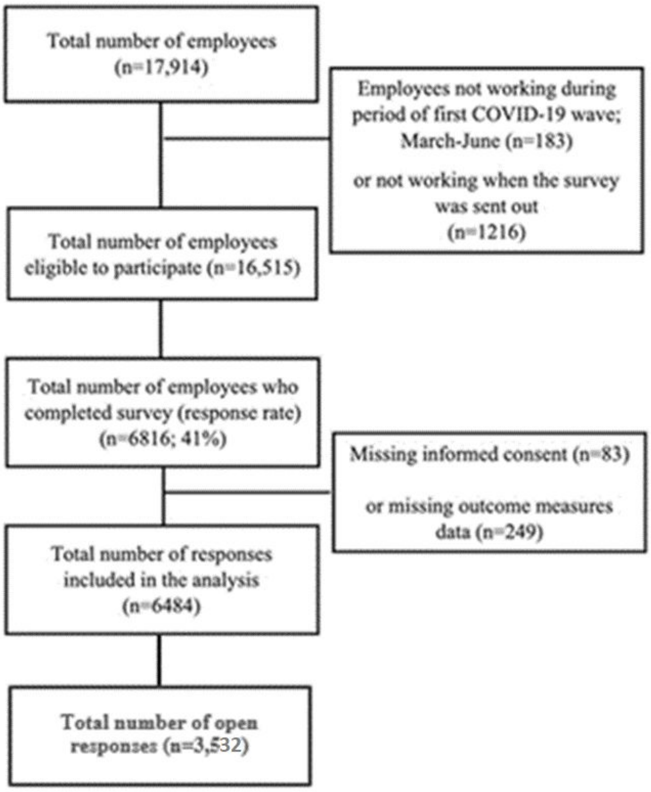

view only

Figure 2. Percentage of healthcare workers who reported a strong worry of being infected or of infecting others during the first wave of the COVID-19 pandemic in Sweden.

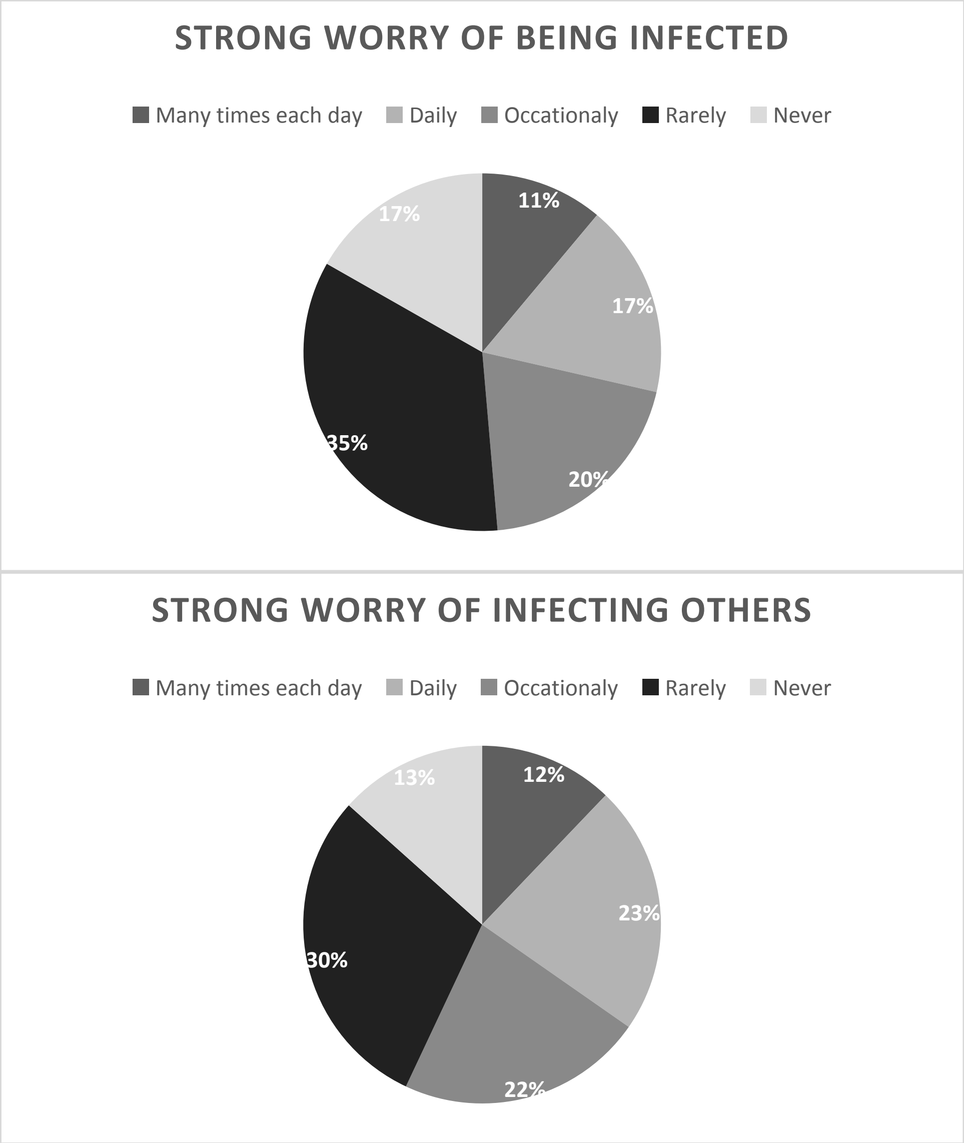

For peer review only - http://bmjopen.bmj.com/site/about/guidelines.xhtml

Figure 3. Percentage of healthcare workers who reported experiencing a strong worry of being infected, stratified by caring/not caring for patients with COVID-19, access/lacking access to personal protective equipment (PPE) when needed, and access/lacking access to emotional support.

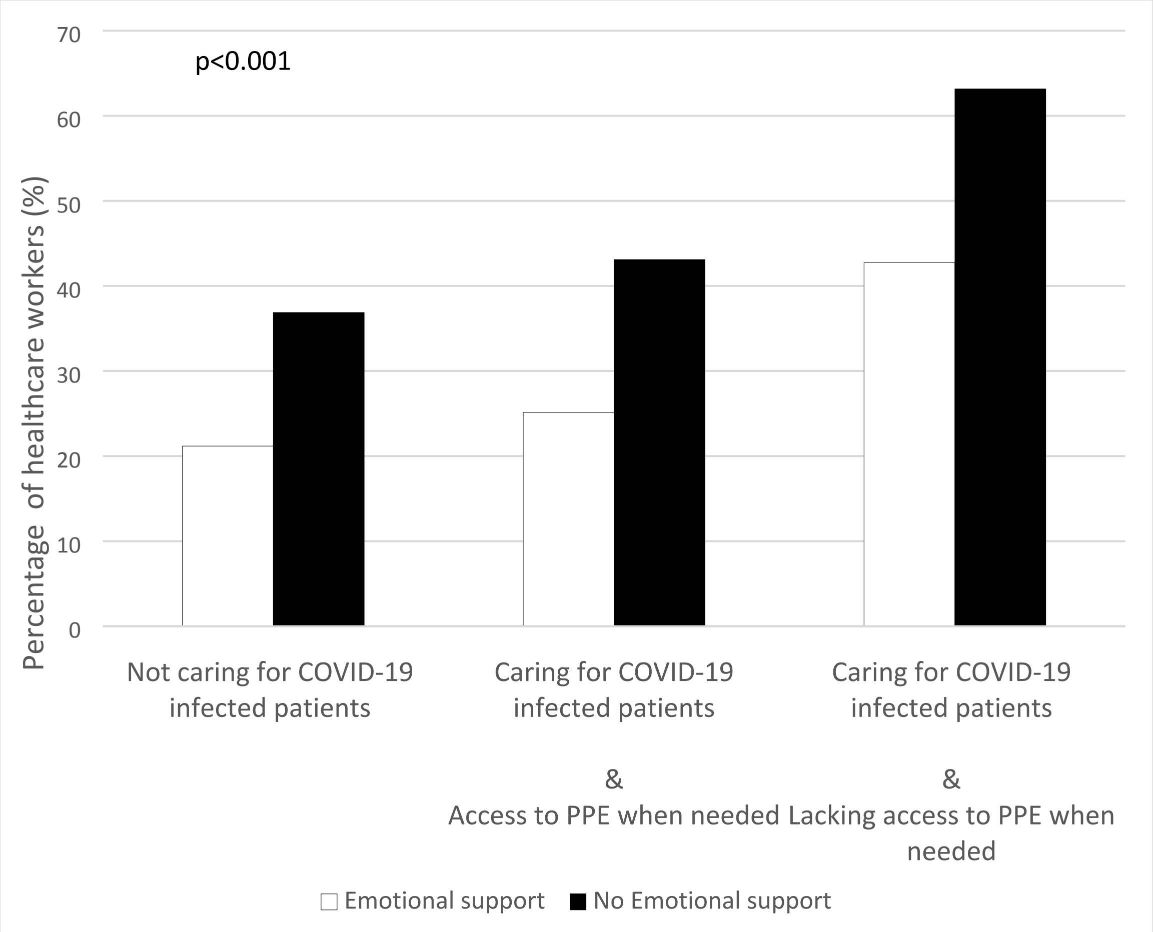

only

For peer review only - http://bmjopen.bmj.com/site/about/guidelines.xhtml

Figure 4. Percentage of healthcare workers who reported experiencing a strong worry about being infected, stratified for caring/not caring for patients with COVID-19, access/lacking access to personal protective equipment (PPE) when needed, and having a reasonable/unreasonable amount of work.

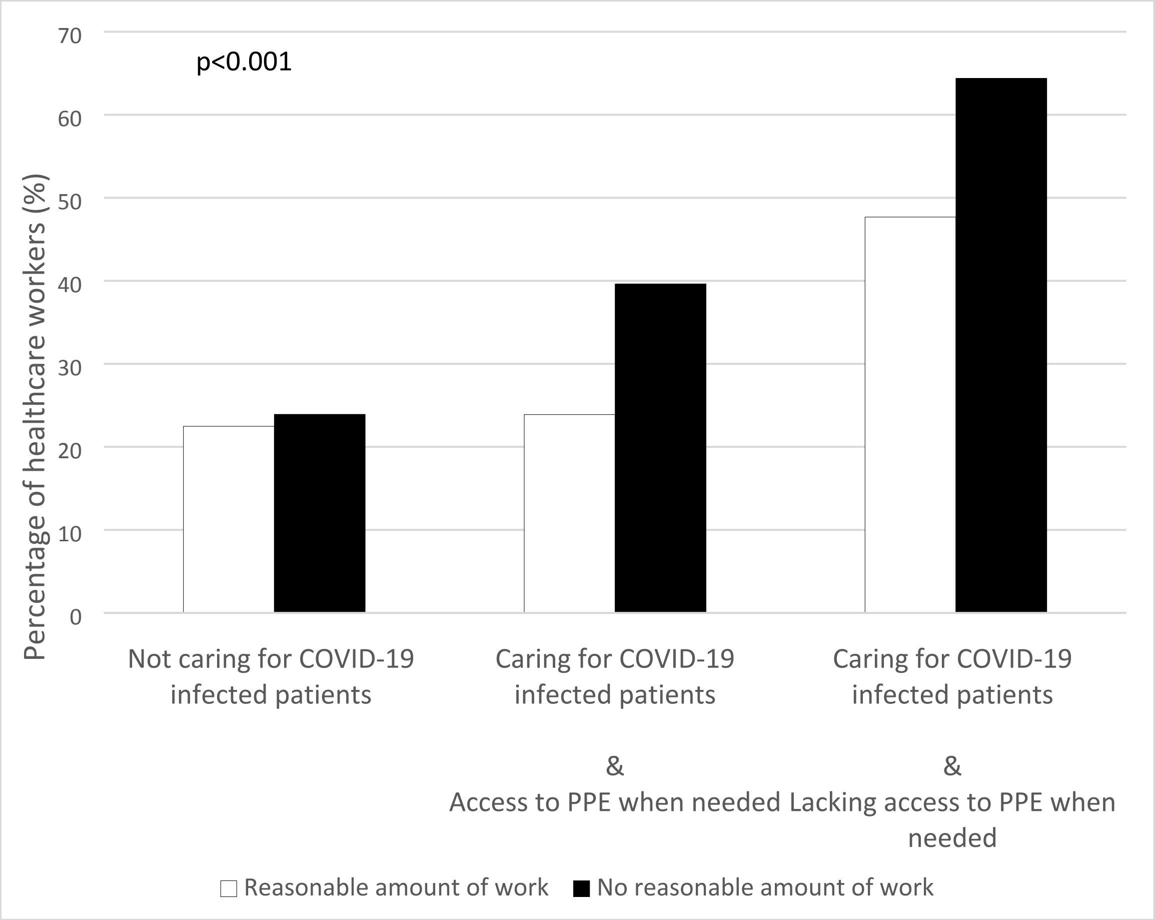

For peer review only - http://bmjopen.bmj.com/site/about/guidelines.xhtml

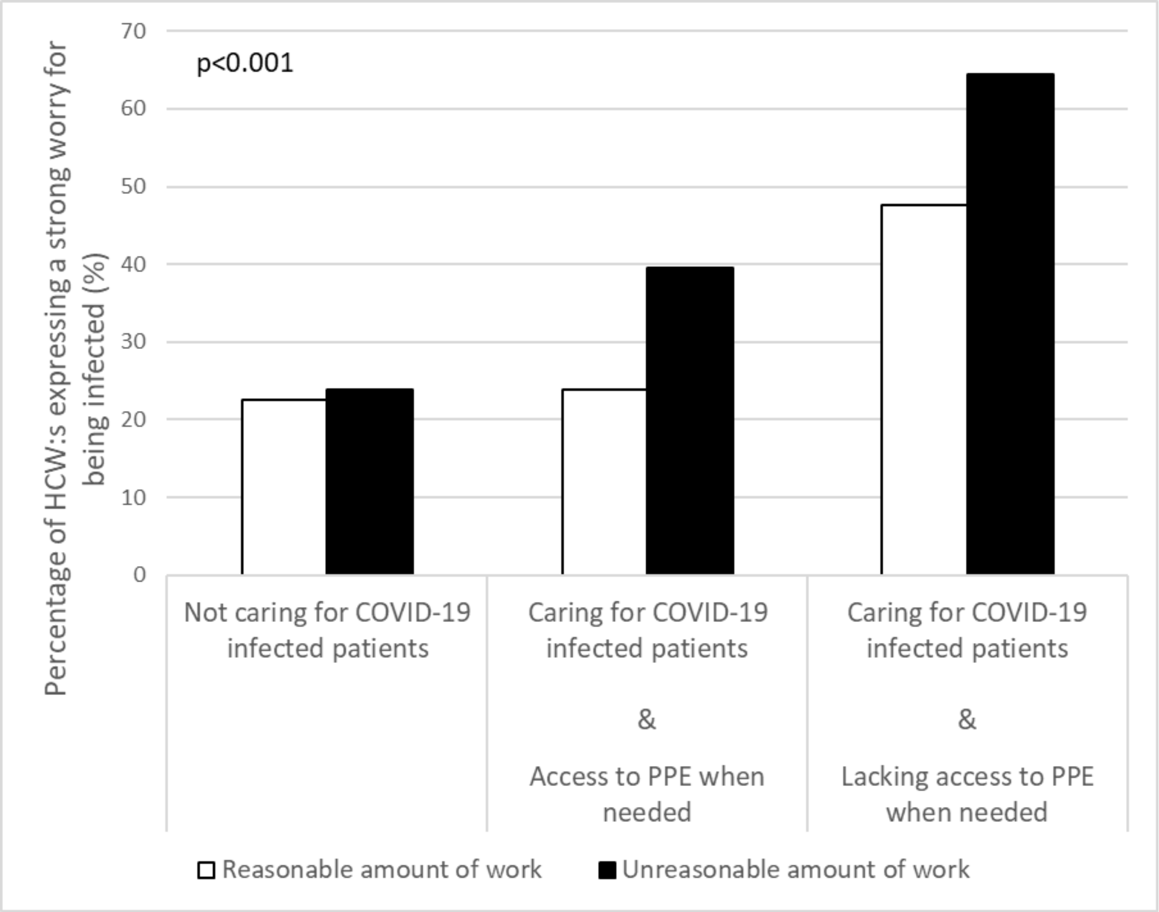

eview only

For peer review only - http://bmjopen.bmj.com/site/about/guidelines.xhtml

BMJ Open

Worry perception and its association with work conditions among healthcare workers during the first wave of the COVID-19 pandemic: a web-based multi-methods survey at a university hospital in Sweden

Journal:	*BMJ Open*
Manuscript ID	bmjopen-2023-080248.R1
Article Type:	Original research
Date Submitted by the Author:	05-Jan-2024
Complete List of Authors:	Alexiou, Eirini; Sahlgrenska University Hospital, Department of Forensic Psychiatry; University of Gothenburg<br>Wijk, Helle; University of Gothenburg; Sahlgrenska University Hospital, Department of Quality Strategies<br>Åkerström, Magnus; Institute of Stress Medicine<br>Jonsdottir, Ingibjörg; Institute of Stress Medicine<br>Degl' Innocenti, Alessio; Region Västra Götaland, Sahlgrenska University Hospital<br>Ahlstrom, Linda; University of Gothenberg
**Primary Subject Heading**:	Occupational and environmental medicine
Secondary Subject Heading:	Occupational and environmental medicine
Keywords:	COVID-19, Health Workforce, OCCUPATIONAL & INDUSTRIAL MEDICINE

SCHOLARONE™
Manuscripts

I, the Submitting Author has the right to grant and does grant on behalf of all authors of the Work (as defined in the below author licence), an exclusive licence and/or a non-exclusive licence for contributions from authors who are: i) UK Crown employees; ii) where BMJ has agreed a CC-BY licence shall apply, and/or iii) in accordance with the terms applicable for US Federal Government officers or employees acting as part of their official duties; on a worldwide, perpetual, irrevocable, royalty-free basis to BMJ Publishing Group Ltd ("BMJ") its licensees and where the relevant Journal is co-owned by BMJ to the co-owners of the Journal, to publish the Work in this journal and any other BMJ products and to exploit all rights, as set out in our licence.

The Submitting Author accepts and understands that any supply made under these terms is made by BMJ to the Submitting Author unless you are acting as an employee on behalf of your employer or a postgraduate student of an affiliated institution which is paying any applicable article publishing charge ("APC") for Open Access articles. Where the Submitting Author wishes to make the Work available on an Open Access basis (and intends to pay the relevant APC), the terms of reuse of such Open Access shall be governed by a Creative Commons licence – details of these licences and which Creative Commons licence will apply to this Work are set out in our licence referred to above.

Other than as permitted in any relevant BMJ Author's Self Archiving Policies, I confirm this Work has not been accepted for publication elsewhere, is not being considered for publication elsewhere and does not duplicate material already published. I confirm all authors consent to publication of this Work and authorise the granting of this licence.

For peer review only

Worry perception and its association with work conditions among healthcare workers during the first wave of the COVID-19 pandemic: a web-based multi-methods survey at a university hospital in Sweden

Eirini Alexiou[1,2*], Helle Wijk[3,4,5], Magnus Akerstrom[6,7], Ingibjörg H. Jonsdottir[6,7], Alessio Degl' Innocenti[2,8], Linda Ahlstrom[3,9]
[1]Department of Forensic Psychiatry, Sahlgrenska University Hospital, Region Västra Götaland, Gothenburg, Sweden
[2]Center for Ethics, Law, and Mental Health (CELAM), Sahlgrenska Academy, University of Gothenburg, Gothenburg, Sweden
[3]Institute of Health and Care Sciences, Sahlgrenska Academy, University of Gothenburg, Gothenburg, Sweden
[4]Department of Quality Strategies, Sahlgrenska University Hospital, Region Västra Götaland, Gothenburg, Sweden
[5]Department of Architecture and Civil Engineering, Chalmers University of Technology, Gothenburg, Sweden
[6]Institute of Stress Medicine, Region Västra Götaland, Gothenburg, Sweden
[7]School of Public Health and Community Medicine, Institute of Medicine, Sahlgrenska Academy, University of Gothenburg, Gothenburg, Sweden
[8]Regionhälsan, Region Västra Götaland, Gothenburg, Sweden
[9]Department of Orthopaedics, Sahlgrenska University Hospital, Region Västra Götaland, Gothenburg, Sweden

*** Correspondence to:**
Eirini Alexiou
eirini.alexiou@vgregion.se

Keywords: COVID-19; worry perceptions; pandemics; content analysis; healthcare workers (HCWs)

Abstract
Objectives In this study, we explored healthcare workers' (HCWs') worry perception and its association with their work situation during the first wave of the COVID-19 pandemic.
Design A web-based multi-methods survey including multiple choice and open-ended questions was used.
Setting The study was conducted at a university hospital in Sweden.
Partcipants All HCWs who were working during the first wave of the COVID-19 pandemic in March-June 2020 were eligible. HCWs (n=6484, response rate 41%) from 69 departments fulfilled the study inclusion criteria and responded to the survey. Of them, we analized data from the 3532 participants who replied to the open-ended questions (54% of the respondents).
Main outcomes measures Worry perception and its association with work conditions among HCWs.
Results 29% (n=1822) and 35% (n=2235) of the responding HCWs experienced a daily, or more than daily, strong worry of being infected or infecting others with the severe acute respiratory syndrome-related coronavirus (SARS-CoV-2). This finding could be further confirmed and explored with themes from the qualitative results: "Ambiguity of feeling safe

For peer review only - http://bmjopen.bmj.com/site/about/guidelines.xhtml

and secure", "Being obliged to adapt to a new reality", and "Into the unknown". The themes consisted of six main categories and 15 sub-categories. The findings revealed that the two main drivers of worry perceived by HCWs were lack of personal protective equipment and fear of bringing the virus home to families and friends.

Conclusions Worries of getting infected are common among HCWs during a crisis such as the COVID-19 pandemic. Several factors are raised that plausibly could minimize the negative effects of worry among the HCWs. Thus, effective preventive work plans should be created, promoted and communicated in order to minimise the effects of such crises and to support HCWs. By focusing on effective communication, preparedness—including access to relevant protective equipment—and general support to the HCWs, the work environment and patient care could be sustained during a crisis such as the COVID-19 pandemic.

Strengths and limitations of this study

- The key strength of this study is transdisciplinary research cooperation, as several multi-disciplinary researchers participated in the implementation of the study and the analysis and interpretation of the data and findings; additionally, use of the NVivo software program enhanced the quality of the analysis, while data were more manageable and ordered, and it was easy to compare and discuss the categories and come to a consensus.
- The pandemic data collection was conducted during a relatively calm period between the first and second waves, which started around November 2020 in Sweden.
- One study limitation is that the quantitative data was cross-sectional, preventing the drawing of conclusions about cause and effect relationships between variables.
- The single-hospital setting and the relatively low response rate limit the generalisability of the findings.
- Another limitation was that, due to the pandemic restrictions, the research methodolgy exclusively utilized written answers and no face-to-face interviews were conducted; this approach presented challenges in facilitating dynamic interaction with participants, as researchers could not ask follow-up questions to further develop and explore the answer.

INTRODUCTION

The COVID-19 pandemic ment a tremendous increase in workload for HCW thereby exacerbating pressure on an already strained workforce and causing additional psychological distress (1).Worry refers to the thoughts, images, emotions, and actions of a negative nature in a repetitive, uncontrollable manner that result from a proactive cognitive risk analysis made to avoid or solve anticipated potential threats and their potential consequences (2). In other words, worry is a natural response to anticipated future problems (3). However, when the threat is uncertain and continuous or when worry is not well calibrated to the actual threat, as during the coronavirus disease (COVID-19) pandemic, worry can become excessive and burdensome (4). The COVID-19 outbreak in China in December 2019 and Europe in February 2020 caused widespread worry, which when at healthy levels led to adaptive, protective behavioral changes. However, for some individuals, the pandemic outbreak led to excessive, maladaptive levels of worry. Experienced worries have been shown to be mainly related to perceived risks for loved ones, possible health problems related to COVID-19, and perceived social restrictions during the pandemic (5). High levels of worry during the COVID-19 pandemic were indicated in several surveys (6, 7). In a cross-sectional study conducted in Thailand, 100% of the included healthcare workers (HCWs) reported worry and fear of COVID-19 related to infection control practices (8). Another cross-sectional study found that HCWs

For peer review only

For peer review only - http://bmjopen.bmj.com/site/about/guidelines.xhtml

reported high levels of worry about getting infected and developing COVID-19, which indicates the great extent to which HCWs were highly worried about this scenario (9). In a cross-sectional study involving healthcare workers, a high prevalence of depression, anxiety, and stress (77-57%) was observed, workplace worries, including concerns about personal infection, transmission to others, inadequate PPE, and insufficient training in PPE usage, were identified as contributing factors (10). This underline the need to address the likely mental health and linked retention crisis in the health care workforce and provide support to create a psychologically safe working environment.

Both frontline and non-frontline HCWs are at risk of severe acute respiratory syndrome-related coronavirus 2 (SARS-CoV-2) infection and may therefore percive significant worries. Early on during the pandemic, COVID-19-pandemic-associated mental health distress became a notable problem for frontline HCWs (11, 12) and a great number of HCWs became infected (13, 14). Frontline HCWs, who took care of patients with COVID-19 daily, were at higher risk of developing worries compared to HCWs who did not participate in the treatment of patients (15). A systematic review report that increased level of potential exposure during the COVID-19 pandemic, as well as in previous coronavirus outbreaks, were shown to cause long-term worries (16). Despite this, the importance of providing support in terms of guide-lines, policy documents and sufficient resoursces to HCW during and after the pandemic to ensure the future of health care staff, have been neglected (1).

In a previous study, we investigated the effects of the COVID-19 pandemic on the working environment of HCWs compared to the pre-pandemic situation (17). Results showed that worries about getting infected was one of several factors that were associated how HCWs perceived their work situation. Thus, the rationale of this study was to extend this knowledge by exploring HCWs' worry perception and the association of worry with perceived working conditions, such as work load and emotional support, during the first wave of the COVID-19 pandemic in Sweden. Understanding factors related to HCWs' worries could be used to design, promote and communicate preventive measures to help alleviate the adverse impacts of such worries to support HCW in similar future situations and thereby improve HCWs' working conditions and counteract the risk of severe distress and burnout.

METHODS

This study is part of a larger research project investigating how the work environment and health of HCWs were affected by the COVID-19 pandemic. The current study utilizes a multi-method design comprising both qualitative and quantitative data from a survey containing multiple choice and open-ended answers. Thus, the survey data with fixed questions was used to analys self-reported quantitative data regarding worry about being infected and infecting others. The fixed survey questions were also used to analys which factors were related to the reported level of worryusing using the STROBE cross sectionalreporting guidelines (18). The open-ended question was used to analys the participants' perceptions related to worry about being infected and infecting others.

Setting

The study was conducted at a large university hospital in northern Europe, situated in western Sweden. The hospital provides emergency and basic care for the more than one million inhabitants in the region and offers highly specialized care for the 1.7 million inhabitants of West Sweden. During the pandemic, the hospital provided intensive care for patients with symptomatic COVID-19 disease.

Population and procedure

For peer review only - http://bmjopen.bmj.com/site/about/guidelines.xhtml

A web-based COVID-19 survey was administered to all hospital HCWs (n=17,914) regardless of having contact with patients (COVID-19 or in general) or having non-clinical work tasks (18). After excluding employees who were absent from work during the study period (n=1,399), 16,515 were eligible for study participation of which 6816 responded to the survey resulting in a response rate of 41% (Figure 1).

During the first week of September 2020, an invitation to participate was sent by e-mail including a link to an anonymous survey. One reminder was provided during the last week of September 2020. The possibility to answer the survey was approximately 5 weeks. Study participants were asked to keep in mind how they perceived their working conditions during the intensive period of the pandemic in the spring of 2020 when answering the questionnaire.

Ethical considerations

The study was approved by the Swedish Ethical Review Authority (ref. 2020-04771) and participants provided informed consent. The study was conducted in compliance with the Helsinki Declaration and the General Data Protection Regulation (EU) 2016/679.

Survey and outcome measures

The survey was designed to be completed in 10-20 minutes. Demographic items included age, gender, organizational affiliation, professional role, specialist training, working hours (daytime, evening, night shifts, or varied hours), and eleven single-items regarding work conditions addressing work demands, support, recovery, and engagement were collected (17). Additional items about work placement during the pandemic, worries about getting infected, and access to personal protective equipment (PPE) were included. Participants were additionally asked to reflect in an open-ended question about their positive and negative experiences of the pandemic during the first wave. The Multi-method research involved the use of two complementary datasets and anaylizing approaches in parallel, guided by a shared overarching research aim (19).

Qualitative analysis

The qualitative part to capture new insights and knowledge of the phenomenon of worry of HCWs during the first wave of COVID-19, we used a qualitative inductive content analysis of the response to the open-ended question, performed in three phases: 1) preparation phase, 2) organizing phase, and 3) reporting phase (20, 21). In the first phase, the first and last authors carefully read the text separately, to obtain their own comprehensive understanding. Thereafter, they each selected the suitable unit of analysis, data that aligns with the intended focus, to enhance credebility and thereafter condensed the data. In the second phase, data were coded, grouped, and sub-categories, main-categories, and themes were created and discussed until consencus by EA, LA and further abstracted with HW. Data were compared for similarities, differences, and patterns, to enhance the confirmability of the findings. The data analysis included an iterative process, with regular reconciliations and discussions, with the larger research group. Working strategically prevented the researcher bias and ensured data coding was externally heterogeneous and internally homogenous. Computer-assisted qualitative data analysis software, Nvivo 12 (QSR International), was used to aid in data organization. Trustworthiness was ensured by following the steps from the data collection to the reporting of the results as stated by Elo *et al.* (21). Analysis also followed the Consolidated criteria for reporting qualitative research (COREQ), a 32-item checklist for interviews (22).

Quantitative analysis

The fixed survey questions were used to analyse the prevalence of perceived worries among the participants and which factors affected the level of worry among the reponders. Individuals without informed consent (n=83), missing data on all work environment items (n=21), or missing an organizational affiliation (n=211) were not included in the analysis. Excluded individuals were evenly distributed among departments and professional roles. One

For peer review only - http://bmjopen.bmj.com/site/about/guidelines.xhtml

administrative department had limited respondents (n=7) and was excluded due to the risk of identification of individuals. The percentage of responding HCWs with a strong worry for being infected and for infecting others during the first wave of the COVID-19 pandemic was summarized descriptively for all survey respondents.

To assess the impact of age, experiencing lack of PPE, being redeployed to another department, caring for patients with COVID-19, and perceived working conditions on HCWs worry of being infected or infecting others, mixed-effects models (Proc Mixed in SAS version 9.4; SAS Institute, USA) were used with these measures as fixed effects (nested within departments) and departments as a random effect. Hypothesis testing for fixed and random effects was performed using Wald tests and likelihood ratio tests, respectively. Statistical significance was set at $p<0.05$ and two-sided confidence intervals were used.

Patient and public involvement

None.

RESULTS

There were 6,484 HCWs, from 69 departments at the hospital, who responded the survey and fulfilled the inclusion criteria for this study. Of them, 3,532 (54%) replied to the open-ended questions, which generated about 216 pages of A4 text.

Most of the responders were women (83%, 5348) and 30% (n=1924) were employed as registered nurses, 21% (n=1327) as assistant nurses, 10% (n=10) as physicians, 12% (n=803) as administrative staff, and the remaining (27%, n=1502) belonged to other occupations such as midwives, psychologists, managers, technicians, and physiotherapists. The age distribution was 12% (n=746) of participants who were ≤ 29 years old, 22% (n=1418) aged 30-39 years, 23% (n=1482) aged 40-49 years, 27% (n=1766) aged 50-59 years, and 16% (n=1045) who were ≥ 60 years.

Percentage of responding HCWs with a strong worry about being infected and infecting others during the first wave of the COVID-19 pandemic

A total of 28% (n=1822) and 35% (n=2235) of the responding HCWs experienced a daily, or more than daily, strong worry about being infected or infecting others, respectively (Figure 2). There was a high correlation between expressing a strong worry about being infected and about infecting others ($r=0.63$, $p<0.001$, $n=6357$).

HCWs worries during the first wave of the COVID-19 pandemic - qualtative analysis

The analysis of the qualitative data resulted in three themes: "Ambiguity of feeling safe and secure", "Being obliged to adapt to a new reality", and "Into the unknown". The themes consisted of a total of six main categories and 15 sub-categories (Table 1).

***Theme:* Ambiguity of feeling safe and secure**

This theme contains conceptions relating to the perceived concern of protection against the virus when exposed. This includes having or lacking access to PPE against COVID-19 such as face masks and/or respirators, face shields/goggles, and protective clothing, as well as conceptions about being able to use the available PPE, and being able to be tested for SARS-CoV-2 when experiencing symptoms. The theme consists of two main categories, "Accessibility" and "Communication", which are both related to reasons the HCWs perceived their ability to protect themselves and patients from encountering the virus as either good or poor.

The main category, "Accessibility", covers three subcategories: availability of PPE and testing for SARS-CoV-2 infection, priorities over testing and using PPE, and training in the proper use of PPE. HCWs described feeling forced to prioritize PPE use when the availability of PPE was limited. In many cases, HCWs lacked access to PPE when needed. Furthermore, they sometimes lacked the opportunity to train and become confident in the proper use of PPE, including when to use it. Results demonstrated that HCWs did not always lacked access to PPE

For peer review only

For peer review only - http://bmjopen.bmj.com/site/about/guidelines.xhtml

and testing due to material shortage but also due to prioritization decisions and to different time schedules and priorities between departments. In general, HCWs experienced that those who were not working in the frontline, were not always allowed to use PPE or be tested for SARS-CoV-2 infection.

“The positive has been that there is protective equipment to use and that all patients are tested regularly.” – P33, Assistant nurse, Geriatrics department

“The negative was that I experienced a few re-evaluations of the need for protective equipment when it started to run out. It was in the middle of that period when we had the most to do and no one knew how it would end. To then claim that COVID-19 was not contagious if you had a visor (which happened to be the protective equipment we had in stock and could be reused) felt disrespectful and completely illogical.” – P66, Medical resident, Geriatrics department and Emergency department

The second main category, “Communication”, contains conceptions about how clarity in information as well as constantly changing routines and decisions, impacted HCWs' feelings of being able to protect themselves and others from the virus.

“Very unclear sometimes regarding protective equipment, especially at the beginning of the pandemic when routines regarding protective equipment were changed very often, created an uncertainty.” – P2, Registered nurse, Pediatrics department

“Lack of clarity in routines when it came to the use of protective equipment in different situations.” – P42, Registered nurse, Radiology department

***Theme:* Being obliged to adapt to a new reality**

This second theme contains perceptions relating to feelings of inadequacy, despite doing one's best, and feelings of sadness when being incapable of providing sufficiently good care. These feelings also addressed handling the challenges in patient care that came with the COVID-19 pandemic, e.g., to have to prioritize daily patient care and make difficult decisions, while feeling scared and sad about being unable to deliver good quality care to patients.

This theme consists of two main categories, “Competence” and “Mission”. The first main category, “Competence”, covers two subcategories, “Learning” and “Workshifting”. These subcategories are related to the feeling of having relevant clinical knowledge (or not) and the skills needed to perform new tasks at the new working place to which HCWs were assigned during the pandemic.

”Being forced to do a work for which you lack training and experience in a completely new workplace without receiving training and without a work description” – P85, Registered nurse, Intensive care unit

“Workshifting, which we fought for a long time to happen, now happened quickly and smoothly”. – P25, Healthcare administrative secretary, medical secretary, Emergency department

The subcategory “Competence” was also related to the feeling of having had the opportunity to learn the skills needed to fight against the virus but also to adapt to the demands of digital transformation and to adopt new technologies by attending courses and other educational and training programs. HCWs report mixed perceptions of adapting to the new skills and tasks, leading in some cases to increased worries and concern.

“What amazing people who work in health care! To adapt quickly to new guidelines, new care, new workplace, colleagues, etc.” – P146, Assistant nurse, Hand surgery department

The second main category, “Mission”, includes two subcategories, “Care priorities” and “Patient outcomes”. These were mostly related to the outcomes of one’s own personal efforts and the feeling of being able to protect patients from getting infection and care for patients to save their lives, as well as making priorities and having enough time to spend with the patients in need.

For peer review only - http://bmjopen.bmj.com/site/about/guidelines.xhtml

"I felt inadequate towards the patients. I could not always provide as good care as desired due to high workload." – P24, Registered nurse, Infectious diseases department

"The most negative thing was all the patients who died without having someone with them. Neither relatives nor HCWs. It feels hard to have been a part of that. Overall, what was experienced as difficult is that the patients with COVID (or suspected COVID) got stuck, their care was not always good." – P31, Registered nurse, Infectious diseases department

***Theme:* Into the unknown**

The third theme contains conceptions relating to the worries about the COVID-19 pandemic and potential outcomes. It consists of two main categories, "Challenges" and "Social interaction". "Challenges" covers two subcategories, "Infection exposure" and "What tomorrow brings". These subcategories are related to worries about being exposed to the COVID-19, of oneself becoming infected, of transmitting the virus to someone else, or that patients infect each other. The second sub-category describes an emotional state caused by feared, unpredictable consequences on personal somatic health. No one knew who would be infected and the outcome of infection for any particular individual.

"I was surprised how scared I was, I'm not a scared person, but this really scared me, and it didn't feel very safe when some of my colleagues were infected at the departments where they worked. It felt good that the hospital locked its doors and that there were guards." – P21, Administrative assistant, Health and rehabilitation department

The second main category, "Social interaction", covers three subcategories: "Socialising with family and friends", "Interaction with colleagues", and "Patient interaction". Reported worries were related to the emotional state due to feared consequences regarding those with whom one was sociliazing or interacting, thereby affecting the health of loved ones, colleagues, and patients not yet infected.

"Negative: Strong concern that my relatives would get COVID-19." – P104, Assistant nurse, Intensive care unit

Factors affecting the level of worry among responding HCWs

The worry of being infected or infecting others decreased with age ($p=0.002$, $n=6349$ and $p<0.001$, $n=6418$, respectively) and increased when experiencing a lack of PPE ($p<0.001$, $n=3933$ and $p<0.001$, $n=3975$, respectively), being redeployed to another department ($p=0.03$, $n=5909$ and $p=0.05$, $n=5969$, respectively) and when caring for patients with COVID-19 ($p<0.001$, $n=6354$ and $p<0.001$, $n=6420$, respectively).

To further disentangle the effect of the factors identified in the qualitative analyses the associations between worry about being infected and caring for patients with COVID-19 in combination with access to PPE when needed and perceived working conditions (i.e., access to emotional support and a reasonable amount of work) were investigated. Results show that caring for patients with COVID-19, especially in combination with a perceived lack of PPE, was associated with a higher percentage of HCWs reporting a daily strong worry about being infected ($p<0.001$, $n=6164$, Figure 3 and $p<0.001$, $n=6254$, Figure 4). Simultaneously, a lack of emotional support (Figure 3) and a reasonable amount of work (Figure 4) led to higher levels of strong worry.

DISCUSSION

Main findings

The quantitative results showed that four out of five responing HCWs reported high levels of worry, half of them daily, which could be further confirmed and explored with themes from the qualitative results.These worries decreased among participants who were supported by accessible PPE and clear communication about routines and how to protect oneself . Worries related to a lack of feeling safe, due to lack of PPE while working, is in line with results from previous studies (23). The rationale for being worried was related to the rapid spread of

For peer review only - http://bmjopen.bmj.com/site/about/guidelines.xhtml

COVID-19, its strong contagion factor, and lethality in severe cases, without any specific medication for treatment (24).
A review study of the psychological trauma faced by healthcare workers in the ICU during the COVID-19 pandemic reveals primary concerns centered around worries of transmitting the virus to their families, personal health worries, witnessing colleagues contracting the disease, and facing stigmatization from their communities of being contagious (25), same results could be seen in several previous studies (26, 27). Confirming our main category, Social Interaction, that the apprehension primarily revolved around the potential consequences for those individuals with whom one was socializing or interacting which were a great concern.

Nevertheless, this is surprising considering the knowledge within the healthcare sector concerning strategies to educate and comfort HCWs in infection control. In the Swedish healthcare setting, there is a long tradition of a strong focus on adherence to hygiene routinesand knowledge regarding how the spread of infection prevention (28, 29, 30). However, there is evidence that repeated training on the proper use of PPE (31), together with regular updates on COVID-19 pandemic (32), is successful in reducing worry among HCWs. So if the hospital would have offered specific and intense training in infection prevention, regular COVID-19 testing, and contact tracing to HCWs on an early stage, this might have decreased the HCWs' worries regarding unsafety and strengthened the impression of the management supporting and empowering employees by taking such actions. On the other hand, a survey from Thailand found that feelings of worry resulted in higher compliance with PPE and infection prevention practices (8), as well as patient safety risk behavior, thereby improving patient outcomes. Pointing at that managers should proactively seek information from recent evidence, and identify situations causing worries (26) on the most effective ways to support and enhance HCWs' safety in order to alleviate and validate the concerns of HCWs, and promoting a positive workplace environment addressing and alleviating worries (10).

Another recommended strategy for managers, is to focus on early signs of individual HCW's worries and to offer support from psychological services (9) and to work closely with their employees, express empathy, gratitude, and prudence for HCWs' valuable efforts (32), and confirm their worries when the situation is beyond one's control (33).
The increased burden on HCWs during crises needs to be adjusted by relocating internal and external flow capacity, and by training and strengthening the available workforce (31). Which skills and competencies that new crises may demand differs and it is therefore complicated and even impossible to know what may be needed when going into the unknown.
Nevertheless, there are ways for organizations to prepare. To adjust to a new reality there is a need to ensure adequate staffing levels with the right competence and clinical knowledge skills, and with appropriate workload to have the confidence to succeed with new tasks without compromising safety, quality of care, and patient outcomes, even though the individual employee might have to work at a different workplace or undergo workshifting.
Times consumed on different work tasks vary, and the schedule does not take into consideration extra time for donning PPE, extra rest days, maintaining education training, and training new colleagues. As reported here, HCWs adapted and learned new skills promptly, and were proud and impressed by that. For this to work, organizations need to be able to better provide both emergency and planned care during and after crises and balancing structures are needed that strengthen organizational resilience (34). Resilience at the individual level has limited impact unless the work to strengthen the work-place also infiltrates the team and organizational levels. Rangachari and Woods (34) therefore highlight the importance of viewing organizational resilience as a system developed by managers to

For peer review only

For peer review only - http://bmjopen.bmj.com/site/about/guidelines.xhtml

learn from the work of individual employees with a focus on proactivity, adaptation, and recovery.

The higher level of worry among HCWs motivated them to adjust to a new, unpredictable reality. One way to address this is through the implementation of effective risk communication programs to increase awareness of the risks associated with the pandemic and promote the adoption of appropriate preventive measures. Healthcare organizations and policymakers need to prioritize the implementation of effective risk communication strategies to promote safety among HCWs. However, there are ways to be prepared for crises in order to improve the work environment including increase safety, and to accept that it is not a failure to admit that everything is unknown, and that one feels very worried (24).

Developing strategies for managing worries can further support building resilience in the face of uncertainty and hinder the consequences of professional worries on work quality and reduce the negative impact on patient outcomes (35). In line with a multi-center study (4), our study identified that the most frequently reported concern was for others to be infected by SARS-CoV-2 and worry about the health of loved ones. Notably, respondents in the multi-center study expressed various worries, including the impact of COVID-19 on the healthcare system, the economy, society, work loss, and changes in daily routines. Our study supports these findings and demonstrates that worries in all areas were referred to as factors that have an impact on the individual, organizational, and societal levels.

Methodological considerations

An essential step in the qualitative data analysis process involved categorizing the results. This process went beyond merely grouping data that appeared similar or connected. Rather, it involved a deliberate effort to gain a deeper understanding of the phenomena under investigation by classifying the data into meaningful categories (20). In this study, the authors' pre-understandings of the studied phenomena differed; the first, second, and last authors are registered nurses, who participated in the first part of the analysis. The variability in experience can be explained by the professions' respective scopes of practice and competencies in relation to their worries.

Quality criteria and recommendations in the form of checklists have been synthesized by multiple publications for researchers. However, there have been raised concerns that checklists may suppress the diversity and multiplicity of practices within the qualitative paradigm, thus researchers must be aware and open-minded. As an alternative, throughout this study, best practice guidelines were applied to achieve and assess methodological rigor and research quality. Guidelines by Elo and Kyngäs (20) and the SRQR checklist (22) were followed to uphold quality. We used data saturation to describe the achievement of a sufficient sample size, and ethical conduct was maintained throughout the research process. The methodology was acknowledged to be limited by researcher bias, which was minimized through predetermined plans. Multiple sources of data were used to optimize accuracy in data collection and analysis processes, resulting in credible and confirmable results (36).

One study limitation is the relatively low response rate (41% of which 54% replied to the open-ended question) which affect the generalisability of the findings. Low response rates are a common problem in research in general and in this study the response rate may also reflect the strained working conditions for HCW's under the COVID-19 pandemic. The survey was also sent out to all employees regardless of whether they were working directly with COVID-19 patients or not. This may partly explain the low response rate if employees refrained from responding as a result of feeling that the survey was not aimed towards them. In line with this we have earlier noticed a somewhat higher response rate among front-line workers compared with e.g. administrative staff (17).

Another issue that should be raised as a limitation is the generalizability of the results. The study was conducted in a single hospital in Sweden, and it is crucial to keep this in mind when

For peer review only - http://bmjopen.bmj.com/site/about/guidelines.xhtml

interpreting the data. However, we have no reason to believe that this hospital differs significantly from others in terms of the challenges faced by hospital staff during COVID-19. The perception of concerns among hospital staff appears to be consistent across hospitals in the western part of the world.

CONCLUSIONS

Overall, this study raises several factors that plausibly could help minimizing the negative impact of the COVID-19 pandemic on HCWs' worries. Developing strategies for managing worries during crises should be created and promoted in order to support and strengthen HCWs. By focusing on effective communication, preparedness, including access to relevant protective equipment, and general support to the HCWs, the work environment and patient care could be sustained during a crisis such as the COVID-19 pandemic.

Declarations

Ethics approval and consent to participate

The studies involving human participants were reviewed and approved by the Swedish Ethical Review Authority (Ref. 2020-04771). The patients/participants provided their written informed consent to participate in this study.

Competing interests

The authors declare that the research was conducted in the absence of any commercial or financial relationships that could be construed as a potential conflict of interest.
The authors declare that they have no competing interests.

Funding

This research received no specific grant from any funding agency in the public, commercial or not-for-profit sectors.

Contributors

EA and LA designed the study, collected the data, analyzed the qualitative data, and drafted and revised the manuscript. HW designed the study, collected the data, and drafted and revised the manuscript. MA designed the study, collected the data, made the quantitative statistical analyses and revised the manuscript. IJ and AD'I designed the study, collected the data, and revised the manuscript. All authors contributed to the article and approved the submitted version.

Acknowledgements

We would like to provide our sincere thanks to the employees of the University Hospital for their time responding to the survey.We would also like to thank Flora Cassiano and Sandra Pettersson for their valuable help with the administration of the survey and with the data analysis. And lastly, we would also like to thank the Human Resources Department at the University Hospital, particularly Camilla Nilsson and Berit Roos Holmquist, for their collaboration and support in the development of the survey.

Data availability statement

The raw data supporting the conclusions of this article will be made available on reasonable request to the corresponding author, without undue reservation.

References

1. Maben, J., Conolly,A., , Abrams, R., , Rowland, E., , Harris, R., , Kelly, K., , Kent, B.,Couper, K. The Impact of Covid On Nurses (ICON) Survey Research Group, 'You can't walk through water without getting wet' UK nurses' distress and psychological health needs during the

For peer review only - http://bmjopen.bmj.com/site/about/guidelines.xhtml

Covid-19 pandemic: A longitudinal interview study, International Journal of Nursing Studies (2022), doi: https://doi.org/10.1016/j.ijnurstu.2022.104242

2. Borkovec TD. Clinical Psychology: Science and Practice. 2002;9:76–80.
3. Bredemeier K, Berenbaum H. "Intolerance of uncertainty and perceived threat". Behav Res Ther. 2008;46(1):28–38. doi:10.1016/j.brat.2007.09.006. PMID 17983612
4. Mertens G, Gerritsen L, Duijndam S, Salemink E, Engelhard IM. Fear of the COVID-19 (COVID-19): Predictors in an online study conducted in March 2020. J Anxiety Disord. 2020;74:102258. doi: 10.1016/j.janxdis.2020.102258.
5. Sobkow A, Zaleskiewicz T, Petrova D, Garcia-Retamero R, Traczyk J. Worry, Risk Perception, and Controllability Predict Intentions Toward COVID-19 Preventive Behaviors. Front Psychol. 2020;11:582720. doi:10.3389/fpsyg.2020.
6. Asmundson GJG, Taylor S. COVID-19phobia: Fear and the 2019-nCoV outbreak. J Anxiety Disord. 2020;70:102196. doi: 10.1016/j.janxdis.2020.102196
7. McCarthy J. U.S. COVID-19 concerns surge, government trust slides (2020) [Online]. Available at: https://news.gallup.com/poll/295505/COVID-19-worries-surge.aspx
8. Apisarnthanarak A, Apisarnthanarak P, Siripraparat C, Saengaram P, Leeprechanon N, Weber DJ. Impact of anxiety and fear for COVID-19 toward infection control practices among Thai healthcare workers. Infect Control Hosp Epidemiol. 2020;41(9):1093-1094.
9. Puci MV, Nosari G, Loi F, Puci GV, Montomoli C, Ferraro OE. Risk Perception and Worries among Healthcare Workers in the COVID-19 Pandemic: Findings from an Italian Survey. Healthcare (Basel, Switzerland). 2020;8(4):535. doi:10.3390/healthcare8040535
10. Ismail, H., Phan, Y. H., Chandriah, K., Arman, M. R., Mokhtar, N. N., Hamdan, S. A., & Yew, S. Q. (2023). Insights into frontline healthcare workers' mental health amidst COVID-19–Sources of workplace worries and coping strategies. Heliyon, 9(9).
11. Tan B, Chew N, Lee G, Jing M, Goh Y, Yeo L, et al. Psychological Impact of the COVID-19 Pandemic on Healthcare Workers in Singapore. Ann Intern Med. 2020;173(4):317–20. https://doi.org/10.7326/M20-1083
12. Lai J, Ma S, Wang Y, et al. Factors Associated With Mental Health Outcomes Among Healthcare Workers Exposed to COVID-19. JAMA Netw Open. 2020;3(3):e203976. doi:10.1001/jamanetworkopen.2020.3
13. Zhang Y, Ma ZF. Impact of the COVID-19 Pandemic on Mental Health and Quality of Life among Local Residents in Liaoning Province, China: A Cross-Sectional Study. Int J Environ Res Public Health. 2020 Mar 31;17(7):2381. doi: 10.3390/ijerph17072381. PMID: 32244498; PMCID: PMC7177660.
14. WHO. COVID-19 disease (COVID-19) pandemic [Online]. 2020; Available at: https://www.who.int/emergencies/diseases/novel-COVID-19 2019?gclid=EAIaIQobChMI7dXri7j36gIVWCUrCh2lLg2TEAAYASAAEgKwIvD_BwE (Accessed December 12, 2020).
15. Maunder RG, Lancee WJ, Balderson KE, et al. Long-term psychological and occupational effects of providing hospital healthcare during SARS outbreak. Emerg Infect Dis. 2006;12(12):1924–1932. doi: 10.3201/eid1212.060584.
16. Carmassi C, Rossi A, Pedrinelli V, Cremone IM, Dell'Oste V, Stratta P, et al. PTSD in the aftermath of a natural disaster: what we learned from the Pisa-L'Aquila Collaboration Project. J Psychopathol. 2020;26(1):99–106. doi: 10.36148/2284-0249-377
17. Jonsdottir IH, Degl'Innocenti A, Ahlstrom L, Finizia C, Wijk H, Åkerström M. A pre/post analysis of the impact of the COVID-19 pandemic on the psychosocial work environment and recovery among healthcare workers in a large university hospital in Sweden. J Public Health Res. 2021;10(4):2329. doi: 10.4081/jphr.2021.2329. PMID: 34278769; PMCID: PMC8715269.
18. von Elm E, Altman DG, Egger M, Pocock SJ, Gøtzsche PC, Vandenbroucke JP; STROBE Initiative. The Strengthening the Reporting of Observational Studies in Epidemiology (STROBE) statement: guidelines for reporting observational studies. Lancet. 2007 Oct 20;370(9596):1453-7. PMID: 18064739. doi: 10.1016/S0140-6736(07)61602-X.
19. Anguera, M. T., Blanco-Villaseñor, A., Losada, J. L., Sánchez-Algarra, P., & Onwuegbuzie, A. J. (2018). Revisiting the difference between mixed methods and multimethods: Is it all in the name?. *Quality & Quantity*, *52*, 2757-2770.
20. Elo S, Kyngäs H. The qualitative content analysis process. J Adv Nurs. 2008;62(1):107-115. doi: 10.1111/j.1365-2648.2007.04569.x
21. Elo S, Kääriäinen M, Kanste O, Pölkki T, Utriainen K, Kyngäs H. Qualitative content analysis: A focus on trustworthiness. SAGE open. 2014;4(1):2158244014522633.

22. O'Brien, BC., Harris IB, Beckman TJ, Reed DA, Cook DA. Standards for reporting qualitative research: a synthesis of recommendations. Academic Medicine, Vol. 89, No. 9 / Sept 2014
23. The Lancet. COVID-19: protecting health-care workers. Lancet. 2020 Mar 21;395(10228):922. doi: 10.1016/S0140-6736(20)30644-9. PMID: 32199474; PMCID: PMC7138074.
24. Koffman J, Gross J, Etkind SN, Selman L. Uncertainty and COVID-19: how are we to respond? J R Soc Med. 2020;113(6):211-216.
25. Wahlster, S., & Hartog, C. (2022). Coronavirus disease 2019 aftermath: psychological trauma in ICU healthcare workers. Current Opinion in Critical Care, 28(6), 686-694.
26. Ceylan, A., Zirek, E., Akturk, S. O., & Calik, B. B. (2022). An examınatıon of the anxıety states of Turkısh health care workers durıng the COVID-19 pandemıc: a cross-sectıonal study. Revista da Associação Médica Brasileira, 68, 389-394.
27. Muhammad, S., Kumari, G., Kumar, N., Tabassum, R., Khaskheli, M., Abbas, J., ... & LAGHARI, S. H. (2021). COVID-19 pandemic and the healthcare workers-The call of duty. Journal of Research in Pharmacy, 25(6), 937-43.
28. Megeus V, Nilsson K, Eriksson B, Karlsson J, Andersson Erichsen A. Hand hygiene and aseptic techniques during routine anesthetic care - observations in the operating room. Antimicrob Resist Infect Control. 2015;4(5).
29. Megeus V, Nilsson K, Karlsson J, Eriksson BI, Andersson AE. Hand contamination, cross-transmission, and risk-associated behaviors: an observational study of team members in ORs. Aorn J. 2015;102(6):645-e1.
30. Wikstrom E, Dellenborg L, Wallin L, Gillespie BM, Erichsen Andersson A. The Safe Hands Study: Implementing aseptic techniques in the operating room: Facilitating mechanisms for contextual negotiation and collective action. Am J Infect Control. 2019;47(3):251-7.
31. Holthof N, Luedi MM. Considerations for acute care staffing during a pandemic. Best Pract Res Clin Anaesthesiol. 2021;35(3):389–404. doi:10.1016/j.bpa.2020.12.008
32. Hofmeyer A, Taylor R. Strategies and resources for nurse leaders to use to lead with empathy and prudence so they understand and address sources of anxiety among nurses practising in the era of COVID-19. J Clin Nurs. 2021;30(1-2):298-305.
33. Radbruch L, Knaul FM, de Lima L, de Joncheere C, Bhadelia A. The key role of palliative care in response to the COVID-19 tsunami of suffering. Lancet. 2020 May 9;395(10235):1467-1469. doi: 10.1016/S0140-6736(20)30964-8. Epub 2020 Apr 22. PMID: 32333842; PMCID: PMC7176394.
34. Rangachari P, Woods L. Preserving organizational resilience, patient safety, and staff retention during COVID-19 requires a holistic consideration of the psychological safety of healthcare workers. Int J Environ Res Public Health. 2020;17(12):4267. doi:10.3390/ijerph17124267
35. Di Trani M, Mariani R, Ferri R, De Berardinis D, Frigo MG. From resilience to burnout in healthcare workers during the COVID-19 emergency: The role of the ability to tolerate uncertainty. Front Psychol. 2021 Apr 16;12:646435. doi: 10.3389/fpsyg.2021.646435. PMID: 33935905; PMCID: PMC8085585.
36. Johnson JL, Adkins D, Chauvin S. A Review of the Quality Indicators of Rigor in Qualitative Research. Am J Pharm Educ. 2020;84(1):7120. doi: 10.5688/ajpe7120

Table 1. Subcategories, main categories, and themes concerning worry perceptions among healthcare workers during the first wave of the COVID-19 pandemic onset in spring 2020

Subcategories	Main categories	Themes
Availability of personal protective equipment and testing for Covid-19	Accessibility	Ambiguity of feeling safe and secure
Priorities over testing and using protective equipment		
Training in proper use of personal protective equipment		

For peer review only - http://bmjopen.bmj.com/site/about/guidelines.xhtml

Information<br>Routines<br>Decisions	Communication	Being obliged to adapt to a new reality
Learning<br>Workshifting	Competence	
Care priorities<br>Patient outcomes	Mission	
Infection exposure<br>What tomorrow brings	Challenges	Into the unknown
Socializing with family and friends<br>Patient interaction<br>Interaction with colleagues	Social interaction	

For peer review only

FIGURE LEGENDS

Figure 1. Participant flowchart

Figure 2. Percentage of participating healthcare workers who reported a strong worry of being infected or of infecting others during the first wave of the COVID-19 pandemic

Figure 3. Percentage of participating healthcare workers who reported experiencing a strong worry of being infected, stratified by caring/not caring for patients with COVID-19, access/lacking access to personal protective equipment (PPE) when needed, and access/lacking access to emotional support

Figure 4. Percentage of participating healthcare workers who reported experiencing a strong worry about being infected, stratified for caring/not caring for patients with COVID-19, access/lacking access to personal protective equipment (PPE) when needed, and having a reasonable/unreasonable amount of work

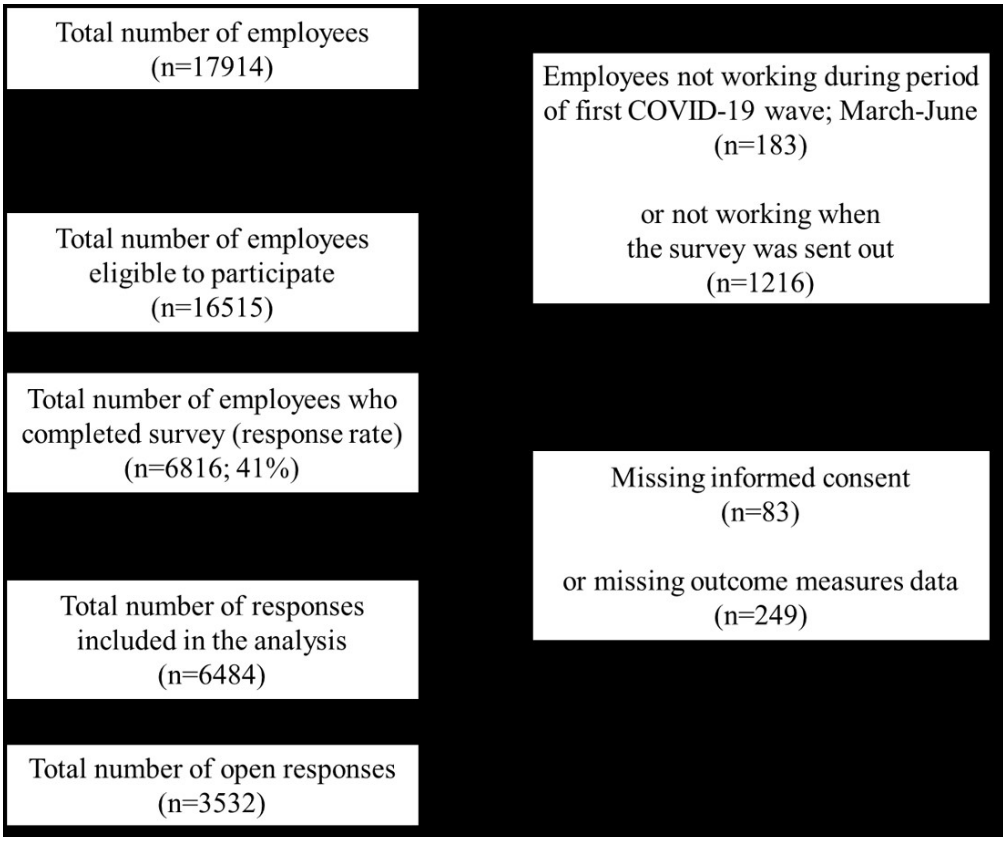

Figure 1. Flow chart showing the total number of hospital health care workers, the number who were eligible to participate, the response rate, and the final number of responses included in the analysis.

185x154mm (150 x 150 DPI)

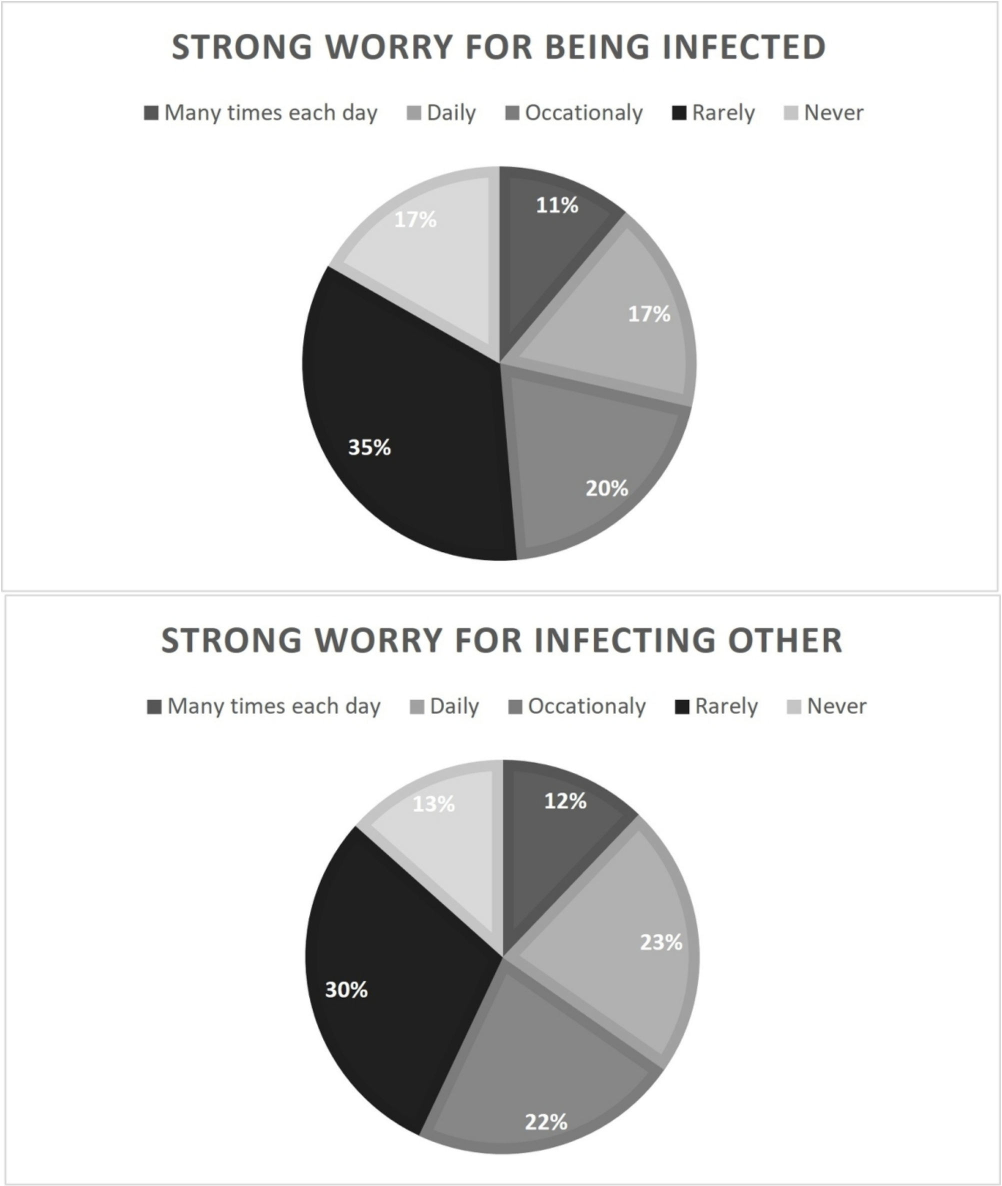

Figure 2. Percentage of healthcare workers who reported a strong worry of being infected or of infecting others during the first wave of the COVID-19 pandemic in Sweden.

127x151mm (300 x 300 DPI)

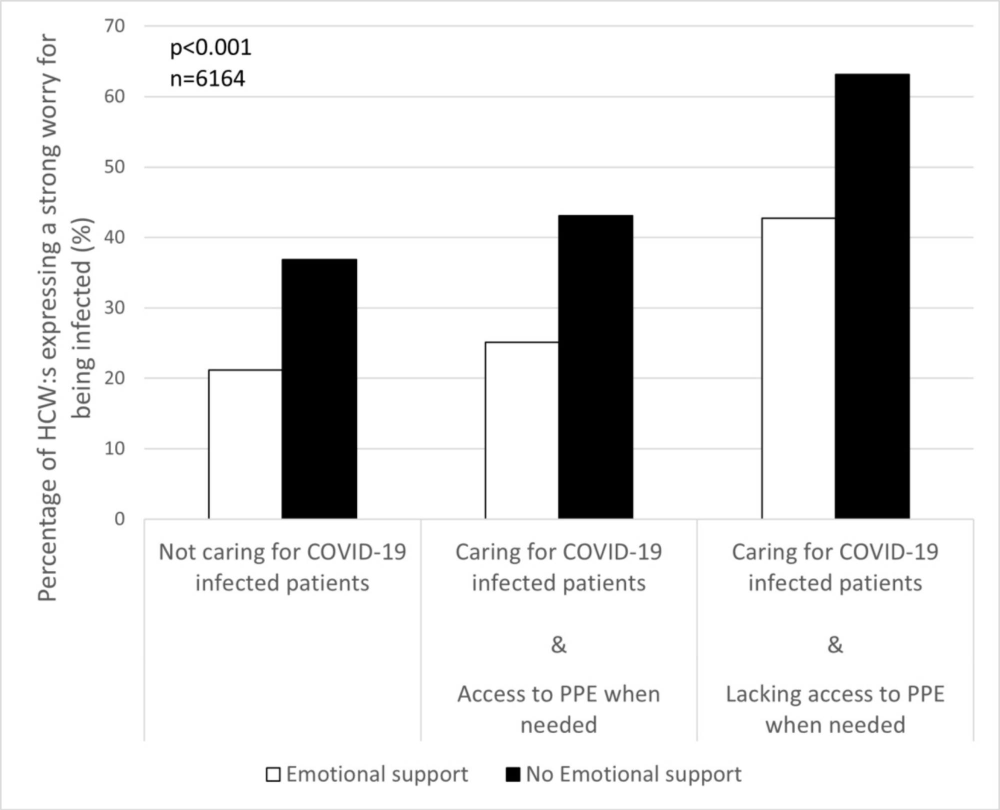

Figure 3. Percentage of healthcare workers who reported experiencing a strong worry of being infected, stratified by caring/not caring for patients with COVID-19, access/lacking access to personal protective equipment (PPE) when needed, and access/lacking access to emotional support.

152x123mm (300 x 300 DPI)

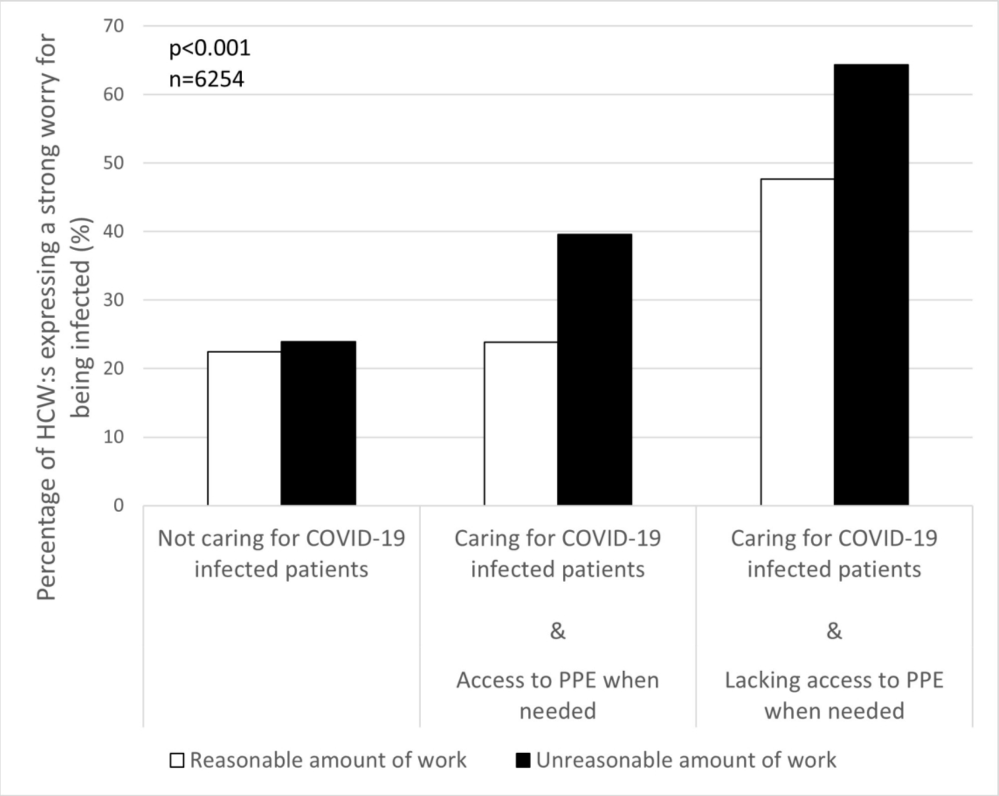

Figure 4. Percentage of healthcare workers who reported experiencing a strong worry about being infected, stratified for caring/not caring for patients with COVID-19, access/lacking access to personal protective equipment (PPE) when needed, and having a reasonable/unreasonable amount of work.

152x121mm (300 x 300 DPI)

Reporting checklist for cross sectional study.

Based on the STROBE cross sectional guidelines.

Instructions to authors

Complete this checklist by entering the page numbers from your manuscript where readers will find each of the items listed below.

Your article may not currently address all the items on the checklist. Please modify your text to include the missing information. If you are certain that an item does not apply, please write "n/a" and provide a short explanation.

Upload your completed checklist as an extra file when you submit to a journal.

In your methods section, say that you used the STROBE cross sectionalreporting guidelines, and cite them as:

von Elm E, Altman DG, Egger M, Pocock SJ, Gotzsche PC, Vandenbroucke JP. The Strengthening the Reporting of Observational Studies in Epidemiology (STROBE) Statement: guidelines for reporting observational studies.

For peer review only

		Reporting Item	Page Number
**Title and abstract**			
Title	#1a	Indicate the study's design with a commonly used term in the title or the abstract	1

Abstract	#1b	Provide in the abstract an informative and balanced summary of what was done and what was found	1-2
**Introduction**			
Background / rationale	#2	Explain the scientific background and rationale for the investigation being reported	2-3
Objectives	#3	State specific objectives, including any prespecified hypotheses	3
**Methods**			
Study design	#4	Present key elements of study design early in the paper	Title & 3
Setting	#5	Describe the setting, locations, and relevant dates, including periods of recruitment, exposure, follow-up, and data collection	3-4
Eligibility criteria	#6a	Give the eligibility criteria, and the sources and methods of selection of participants.	3-4
	#7	Clearly define all outcomes, exposures, predictors, potential confounders, and effect modifiers. Give diagnostic criteria, if applicable	4-5
Data sources / measurement	#8	For each variable of interest give sources of data and details of methods of assessment (measurement). Describe comparability of assessment methods if there is more than one group. Give information separately for for exposed and unexposed groups if applicable.	4-5

For peer review only

Bias	#9	Describe any efforts to address potential sources of bias	n/a
Study size	#10	Explain how the study size was arrived at	3
Quantitative variables	#11	Explain how quantitative variables were handled in the analyses. If applicable, describe which groupings were chosen, and why	4-5
Statistical methods	#12a	Describe all statistical methods, including those used to control for confounding	5
Statistical methods	#12b	Describe any methods used to examine subgroups and interactions	5
Statistical methods	#12c	Explain how missing data were addressed	4
Statistical methods	#12d	If applicable, describe analytical methods taking account of sampling strategy	5
Statistical methods	#12e	Describe any sensitivity analyses	n/a
**Results**			
Participants	#13a	Report numbers of individuals at each stage of study—eg numbers potentially eligible, examined for eligibility, confirmed eligible, included in the study, completing follow-up, and analysed. Give information separately for for exposed and unexposed groups if applicable.	4
Participants	#13b	Give reasons for non-participation at each stage	9

For peer review only

Participants	#13c	Consider use of a flow diagram	Figure 1
Descriptive data	#14a	Give characteristics of study participants (eg demographic, clinical, social) and information on exposures and potential confounders. Give information separately for exposed and unexposed groups if applicable.	3-4
Descriptive data	#14b	Indicate number of participants with missing data for each variable of interest	3, 4
Outcome data	#15	Report numbers of outcome events or summary measures. Give information separately for exposed and unexposed groups if applicable.	4
Main results	#16a	Give unadjusted estimates and, if applicable, confounder-adjusted estimates and their precision (eg, 95% confidence interval). Make clear which confounders were adjusted for and why they were included	5,7
Main results	#16b	Report category boundaries when continuous variables were categorized	5
Main results	#16c	If relevant, consider translating estimates of relative risk into absolute risk for a meaningful time period	n/a
Other analyses	#17	Report other analyses done—e.g., analyses of subgroups and interactions, and sensitivity analyses	7
**Discussion**			
Key results	#18	Summarise key results with reference to study objectives	5

For peer review only

Limitations	#19	Discuss limitations of the study, taking into account sources of potential bias or imprecision. Discuss both direction and magnitude of any potential bias.	1 & 9
Interpretation	#20	Give a cautious overall interpretation considering objectives, limitations, multiplicity of analyses, results from similar studies, and other relevant evidence.	7,10
Generalisability	#21	Discuss the generalisability (external validity) of the study results	8-9
**Other Information**			
Funding	#22	Give the source of funding and the role of the funders for the present study and, if applicable, for the original study on which the present article is based	10

None The STROBE checklist is distributed under the terms of the Creative Commons Attribution License CC-BY. This checklist can be completed online using https://www.goodreports.org/, a tool made by the EQUATOR Network in collaboration with Penelope.ai

For peer review only

Standards for Reporting Qualitative Research (SRQR)*

http://www.equator-network.org/reporting-guidelines/srqr/

Title and abstract

	Page/line no(s).
**Title** - Concise description of the nature and topic of the study Identifying the study as qualitative or indicating the approach (e.g., ethnography, grounded theory) or data collection methods (e.g., interview, focus group) is recommended	P1/ line 1-4
**Abstract** - Summary of key elements of the study using the abstract format of the intended publication; typically includes background, purpose, methods, results, and conclusions	P1-2/ line 30-57

Introduction

**Problem formulation** - Description and significance of the problem/phenomenon studied; review of relevant theory and empirical work; problem statement	P3/line 119-12
**Purpose or research question** - Purpose of the study and specific objectives or questions	P3/ line 119-125

Methods

**Qualitative approach and research paradigm** - Qualitative approach (e.g., ethnography, grounded theory, case study, phenomenology, narrative research) and guiding theory if appropriate; identifying the research paradigm (e.g., postpositivist, constructivist/ interpretivist) is also recommended; rationale**	P3/ line 128-130
**Researcher characteristics and reflexivity** - Researchers' characteristics that may influence the research, including personal attributes, qualifications/experience, relationship with participants, assumptions, and/or presuppositions; potential or actual interaction between researchers' characteristics and the research questions, approach, methods, results, and/or transferability	P4/ line 180-181<br>P9/ line 410-414
**Context** - Setting/site and salient contextual factors; rationale**	P3/ line 136-140
**Sampling strategy** - How and why research participants, documents, or events were selected; criteria for deciding when no further sampling was necessary (e.g., sampling saturation); rationale**	P4/ 143-147
**Ethical issues pertaining to human subjects** - Documentation of approval by an appropriate ethics review board and participant consent, or explanation for lack thereof; other confidentiality and data security issues	P4/ line 153
**Data collection methods** - Types of data collected; details of data collection procedures including (as appropriate) start and stop dates of data collection and analysis, iterative process, triangulation of sources/methods, and modification of procedures in response to evolving study findings; rationale**	P4/ line 169-185

For peer review only - http://bmjopen.bmj.com/site/about/guidelines.xhtml

**Data collection instruments and technologies** - Description of instruments (e.g., interview guides, questionnaires) and devices (e.g., audio recorders) used for data collection; if/how the instrument(s) changed over the course of the study	P4/ line 163-164
**Units of study** - Number and relevant characteristics of participants, documents, or events included in the study; level of participation (could be reported in results)	P5/ line 207-216
**Data processing** - Methods for processing data prior to and during analysis, including transcription, data entry, data management and security, verification of data integrity, data coding, and anonymization/de-identification of excerpts	P4/line 169-186
**Data analysis** - Process by which inferences, themes, etc., were identified and developed, including the researchers involved in data analysis; usually references a specific paradigm or approach; rationale**	P4/line 169-186
**Techniques to enhance trustworthiness** - Techniques to enhance trustworthiness and credibility of data analysis (e.g., member checking, audit trail, triangulation); rationale**	P4/ line 183-185

Results/findings

**Synthesis and interpretation** - Main findings (e.g., interpretations, inferences, and themes); might include development of a theory or model, or integration with prior research or theory	Table 1 /P12-13
**Links to empirical data** - Evidence (e.g., quotes, field notes, text excerpts, photographs) to substantiate analytic findings	The qualitative results are substantiated by quotes, eg P6/line 247-254

Discussion

**Integration with prior work, implications, transferability, and contribution(s) to the field** - Short summary of main findings; explanation of how findings and conclusions connect to, support, elaborate on, or challenge conclusions of earlier scholarship; discussion of scope of application/generalizability; identification of unique contribution(s) to scholarship in a discipline or field	P9/line 390-406
**Limitations** - Trustworthiness and limitations of findings	P9/line 416-426

Other

**Conflicts of interest** - Potential sources of influence or perceived influence on study conduct and conclusions; how these were managed	P10/ line 458-460
**Funding** - Sources of funding and other support; role of funders in data collection, interpretation, and reporting	P10/ line 462-463

*The authors created the SRQR by searching the literature to identify guidelines, reporting standards, and critical appraisal criteria for qualitative research; reviewing the reference lists of retrieved sources; and contacting experts to gain feedback. The SRQR aims to improve the transparency of all aspects of qualitative research by providing clear standards for reporting qualitative research.

For peer review only

For peer review only - http://bmjopen.bmj.com/site/about/guidelines.xhtml

**The rationale should briefly discuss the justification for choosing that theory, approach, method, or technique rather than other options available, the assumptions and limitations implicit in those choices, and how those choices influence study conclusions and transferability. As appropriate, the rationale for several items might be discussed together.

Reference:

O'Brien BC, Harris IB, Beckman TJ, Reed DA, Cook DA. **Standards for reporting qualitative research: a synthesis of recommendations.** *Academic Medicine*, Vol. 89, No. 9 / Sept 2014 DOI: 10.1097/ACM.0000000000000388

For peer review only

For peer review only - http://bmjopen.bmj.com/site/about/guidelines.xhtml
